# Role of Peptides in Skeletal Muscle Wasting: A Scoping Review

**DOI:** 10.1002/jcsm.70109

**Published:** 2025-11-13

**Authors:** Petar Naumovski, Bart De Spiegeleer, Aster Wakjira, Christophe Van De Wiele, Vincent Mouly, Katarzyna Goljanek‐Whysall, Kauê Santana da Costa, Ewerton Cristhian Lima de Oliveira, Evelien Wynendaele, Anton De Spiegeleer

**Affiliations:** ^1^ Translational Research in Immunosenescence, Gerontology and Geriatrics (TRIGG) Group Ghent University Hospital Ghent Belgium; ^2^ Drug Quality and Registration (DruQuaR) Group, Faculty of Pharmaceutical Sciences Ghent University Ghent Belgium; ^3^ Department of Geriatrics, Faculty of Medicine and Health Sciences Ghent University Hospital Ghent Belgium; ^4^ Department of Clinical Pharmacy, School of Pharmacy, Institute of Health Sciences Jimma University Jimma Ethiopia; ^5^ Department of Diagnostic Sciences, Faculty of Medicine and Health Sciences Ghent University Hospital Ghent Belgium; ^6^ Institut de Myologie, Centre de Recherche en Myologie, Inserm Sorbonne Université Paris France; ^7^ Department of Physiology, School of Medicine, Galway RNA Research Cluster NUI Galway Galway Ireland; ^8^ Laboratory of Computational Simulation, Institute of Biodiversity Federal University of Western Pará Santarém Pará Brazil; ^9^ Instituto Tecnológico Vale (ITV) Belém Pará Brazil

**Keywords:** cachexia, muscle wasting, peptides, sarcopenia

## Abstract

**Background:**

Systemic muscle wasting is a prevalent condition that predicts adverse health outcomes in aging and disease. Despite its clinical relevance, the development of predictive biomarkers and effective pharmacological therapies remains limited. Peptides have recently gained attention for their diverse bioactive functions, positioning them as promising biomarkers and therapeutic agents for muscle wasting.

**Methods:**

This scoping review systematically identifies studies examining the direct association between well‐defined peptides and clinical components of muscle wasting: muscle mass, strength and physical performance. The review follows the Preferred Reporting Items for Systematic Reviews and Meta‐Analysis for Scoping Reviews (PRISMA‐ScR) guidelines. A comprehensive search of Embase, PubMed and Web of Science was conducted up to 31 October 2024, focusing on original human or animal studies. Studies involving congenital or inherited muscle disorders, inflammatory myopathies and neurodegenerative diseases, such as Parkinson's disease, were excluded. A snowball approach was used to synthesize the presumed cellular pathways of identified peptides.

**Results:**

A total of 126 studies were included: 71 (56.3%) focused on a single muscle wasting component (48 on mass, 16 on strength and 7 on performance), 31 (24.6%) examined two, 16 (12.7%) analysed all three separately, and 8 (6.3%) assessed sarcopenia as a categorical variable.

Eighty‐seven distinct peptides linked to muscle wasting were identified, ranging from collagen tripeptide (3 amino acids) to insulin (51 amino acids). The most studied peptides are ghrelin (14.3%), brain natriuretic peptide (BNP, 11.1%), C‐peptide (11.1%), insulin (10.3%) and Szeto‐Schiller 31 (SS‐31, 6.3%). Most (62.1%) influence one or more of four key muscle homeostasis pathways (PI3K/Akt/mTOR, ActR/SMAD, IKK/NF‐κB and AMPK/PGC1α), which regulate atrophy (via FOXO, NF‐κB, SMAD2/3, glucocorticoid receptor and GSK‐3β) and hypertrophy (via androgen receptors, PGC‐1α and S6K). Flaws in study design and reporting were prevalent, hindering clinical translation. Sex bias was evident, with females comprising 23.9% of participants in human interventional studies and only 9.1% and 12.4% of mice and rats in rodent studies, respectively. Clinical, pre‐analytical and analytical reporting gaps were common: 56.6% documented diurnal timing, food intake and activity around peptide collection; none specified storage‐to‐analysis duration; and only 11.5% reported detection limits for peptide measurements.

**Conclusion:**

This scoping review highlights the potential of peptides as biomarkers and intervention targets for muscle wasting. It connects the cellular receptors and signaling pathways linking peptides with skeletal muscle wasting. Improving clinical translation requires addressing study design limitations, incorporating more representative study populations and adhering to standardized reporting guidelines. The application of machine learning can support the identification of novel bioactive peptides.

## 1 Introduction

Systemic muscle wasting is marked by an imbalance between protein synthesis and degradation in skeletal muscle, leading to significant muscle mass loss. Various muscle wasting types, including sarcopenia and cachexia, differ in causes, progression rates and biological pathways, although they share core transcriptional programs [1]. Sarcopenia, the accelerated age‐related decline in skeletal muscle mass and strength, affects 10%–27% of individuals aged 60 years or older, with prevalence varying by diagnostic criteria [2]. Given that approximately 15% of the global population is over 60, this translates to 1.5%–4% of the total population being affected [3]. Cachexia, the subacute systemic muscle wasting associated with irreversible malnutrition and weight loss in the context of severe illnesses, such as heart failure, kidney disease and cancer, impacts 0.5%–1.0% of the European population, with prevalence varying from 2% in rheumatoid arthritis to as high as 27% in cancer patients [4].

Muscle wasting is linked to increased all‐cause mortality (RR = 1.36 when low muscle mass) [5], functional disability (OR = 3.03 in sarcopenia) [6], higher hospitalization rates (Incident Rate Ratio = 1.52 when reduced muscle strength) [7] and significant reductions in quality of life (e.g., 40% lower Patient Health Questionnaire‐9 scores in cancer cachexia and 5.4% decrease in Medical Outcomes Study Health Survey 36‐Item scores in sarcopenia) [8]. Also, economically, it imposes a substantial burden; for example, US hospitalizations cost $4000 more for a cachectic patient, whereas a sarcopenic individual incurs an average annual increase of $2316 in hospitalization costs [9].

Peptides, biopolymers composed of 2–50 amino acids, play vital roles in diverse biological processes, including structural stability (e.g., collagen peptides), energy metabolism (e.g., insulin), blood pressure regulation (e.g., angiotensin II), reproduction (e.g., gonadotropin‐releasing hormone or GnRH), social bonding (e.g., oxytocin) and host defence (e.g., defensins) [10]. Their unique structural properties offer advantages in tissue and cell penetration, selectivity, immunogenicity, environmental biodegradability and cost‐effectiveness, making them increasingly attractive as theranostics. However, challenges like limited oral bioavailability and rapid plasma degradation persist. Solutions such as post‐translational or chemical modifications are being explored to address these limitations [11]. With over 120 peptide‐based drugs or diagnostics currently approved, the peptide market is expected to grow significantly, with a projected annual growth rate of 6.3% from 2023 to 2030, reaching an estimated $68.6 billion by 2030 [12].

With the increasing importance of both muscle wasting and peptides, research linking these two areas across diverse disciplines is accumulating. However, fragmentation in this research has hindered comprehensive understanding. This scoping review consolidates current knowledge on peptides associated with muscle wasting in human and animal models. It aims to (1) map the characteristics of studies examining peptides across various muscle wasting conditions, (2) map peptides investigated across these conditions and (3) schematically outline the various peptide‐mediated cellular signalling pathways involved in muscle wasting conditions. By synthesizing findings and identifying knowledge gaps, this review provides a foundation for translating peptide research into clinical practice for muscle wasting.

## 2 Methods

This review was conducted in accordance with the Preferred Reporting Items for Systematic Reviews and Meta‐Analysis (PRISMA) guidelines for scoping reviews (PRISMA‐ScR). The review protocol was not registered in a public database, as protocol registration is currently not universally supported for scoping reviews. The full methodological details are provided in Supporting Information S1.

### 2.1 Research Question

This scoping review examines the role of peptides in muscle wasting, focusing on their in vivo effects, cellular mechanisms and research gaps.

### 2.2 Data Sources and Search Strategy

A systematic search in Embase, PubMed and Web of Science (up to 31 October 2024) included studies on peptides and muscle wasting preclinical models and clinical studies, excluding genetic muscle diseases, inflammatory myopathies and cardiac or smooth muscle research.

### 2.3 Study Eligibility Criteria

Included studies involved humans or animals, assessing peptides linked to muscle function. Proteins, undefined peptide mixtures, in vitro–only studies and neurodegenerative or congenital disease research were excluded.

### 2.4 Screening and Article Selection

Two reviewers screened articles using Rayyan software, resolving conflicts through discussion. The process followed a PRISMA flow diagram.

### 2.5 Data Extraction

Extracted data included study details, peptide characteristics, interventions, outcomes and methods, with disagreements resolved systematically.

### 2.6 Physicochemical Properties

Peptides were analysed for hydrophobicity, binding potential, isoelectric point and stability using computational tools.

### 2.7 Cellular Pathways

Mechanistic insights were synthesized using a snowball approach, mapping peptide–muscle interactions.

## 3 Results

**FIGURE 1:**
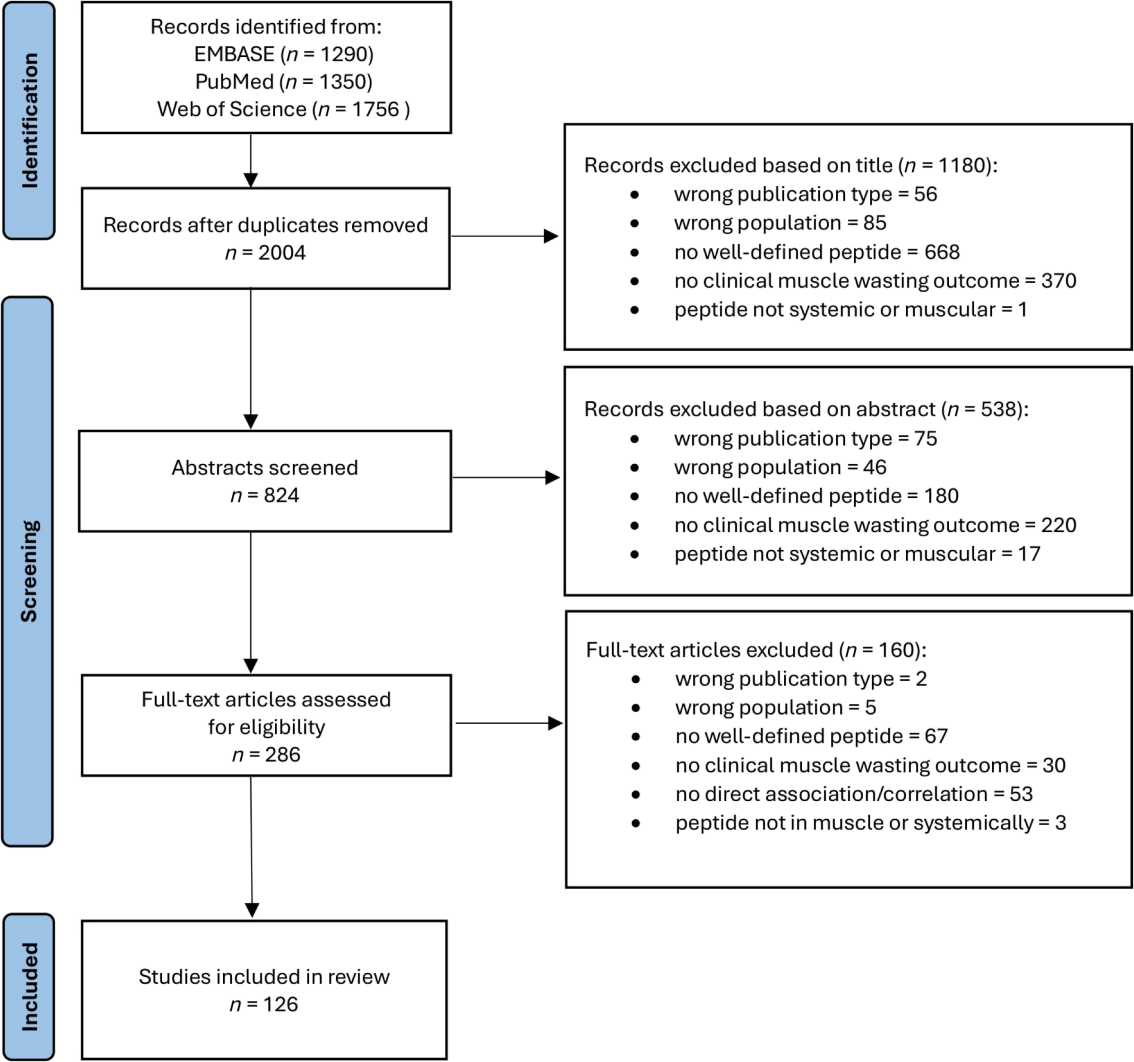
Preferred Reporting Items for Systematic Review and Meta‐Analysis (PRISMA) flow diagram for publication screening.

Overall, our systematic search strategy identified 2004 unique records. After screening titles and abstracts, 1718 records were excluded, leaving 286 studies for full‐text review. Ultimately, 126 records were eligible for this scoping review (flow diagram in Figure 1), with detailed data extraction provided in Supporting Information S2. The most common reasons for exclusion were a lack of well‐defined peptides, meaning studies either did not investigate peptides (but, e.g., proteins) or assessed only peptide mixtures/protein hydrolysates without characterizing specific peptides (915), absence of clinical muscle wasting outcomes (620), wrong population (e.g., Duchenne muscular dystrophy or Parkinson's disease) (136) and wrong publication type (e.g., review) (133).

### 3.1 Study Characteristics

**FIGURE 2:**
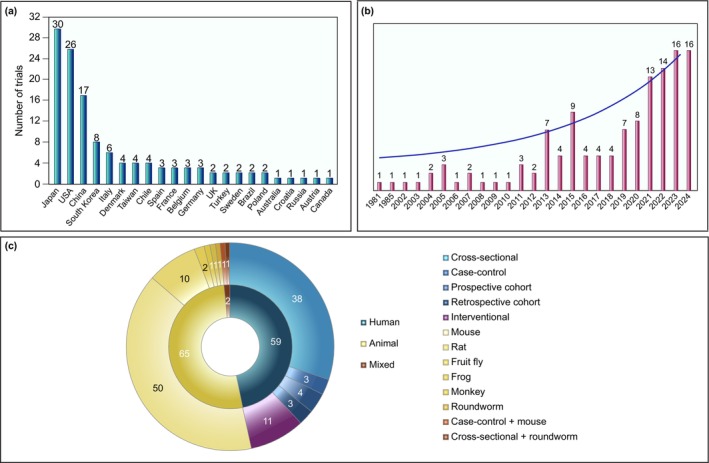
General characteristics of the included studies. (A) Distribution of studies according to the countries in which the study was conducted. (B) Bar graph showing the exponential growth in the number of studies per year (fitted function in blue). (C) Donut chart illustrating the distribution of study design types. The inner circle shows the overall number of human, animal and mixed studies, whereas the outer circle shows the specific study design or animal order/genus for human and animal studies, respectively.

The 126 included studies originate from 22 different countries (Figure 2A), with the majority coming from Japan (23.8%) and the USA (20.6%). There is an exponential increase in the number of reported studies per year, with more than half of them published in 2018 or later (Figure 2B); 65 (51.6%) of the studies are animal studies, 59 (46.8%) are human studies (38.1% human observational studies and 8.7% human interventional studies), and 2 studies (1.6%) contain both an animal and human observational component (mice/human case–control and roundworm/human cross‐sectional). Among the animal studies, 51 (76.1%) are conducted on mice, 10 (14.9%) on rats, 2 (2.9%) on fruit flies and roundworms and 1 (1.5%) on frogs and monkeys. The human observational studies comprise 38 (79.1%) cross‐sectional, 4 (8.3%) prospective cohort, 3 (6.2%) case–control and 3 (6.2%) retrospective cohort studies (Figure 2C). Most animal studies last between a few days and a few weeks, with the shortest being 2 days [13] and the longest 9 months [14]. Human observational studies range from 25 days [15] to 16 years [16], whereas the duration of human interventional studies varies from 1 day [17] to 2 years [18].

**FIGURE 3:**
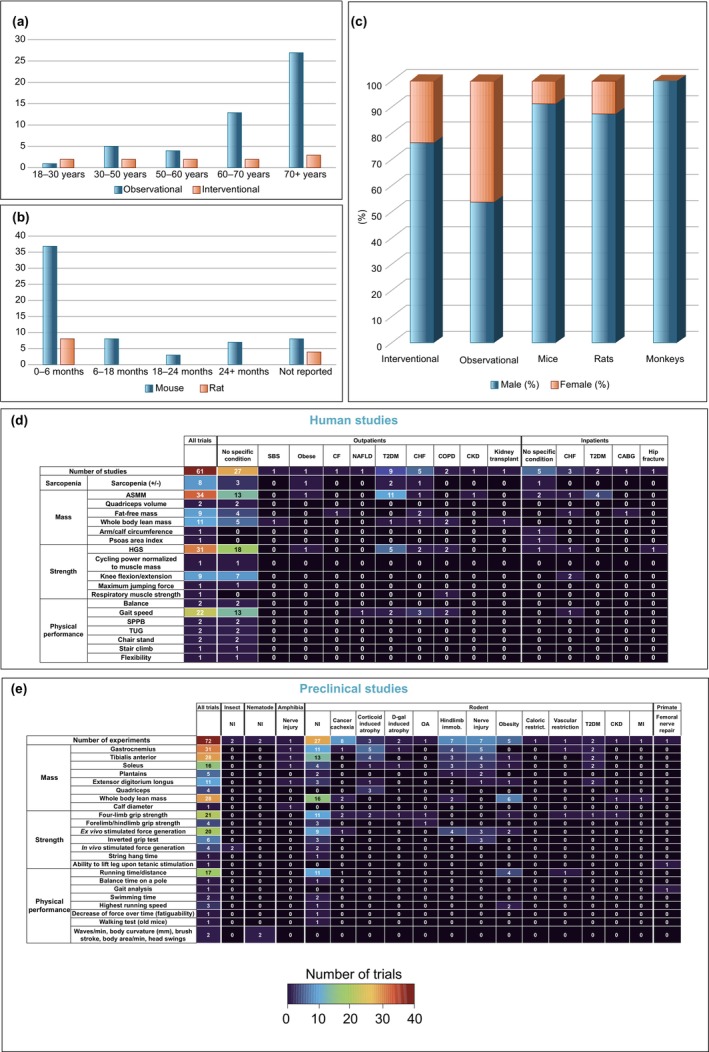
Graphical summary of the population characteristics (A) Bar graph showing the distribution of population age across human studies (*y*‐axis: number of studies). (B) Bar graph showing the distribution of the population age across rat and mouse studies (*y*‐axis: number of studies). (C) Bar graph depicting the distribution of sexes across human studies and animal models. (D) Heat map illustrating the human population characteristics including various clinical conditions in which distinct skeletal muscle clinical endpoints were measured (Low values: blue, high values: red). Sarcopenia diagnosis was based on EWGSOP 1/2 or AWGS criteria. Separate outcome components used to diagnose sarcopenia are also included in the numbers. ASMM includes both measurements normalized and not normalized to height squared (ASMI). ASMM: appendicular skeletal muscle mass, CABG: coronary artery bypass graft, CF: cystic fibrosis, CHF: chronic heart failure: CKD: chronic kidney disease, COPD: chronic obstructive pulmonary disease, HGS: handgrip strength, NAFLD: non‐alcoholic fatty liver disease, SBS: short bowel syndrome, SPPB: short physical performance battery, T2DM: Type 2 diabetes mellitus, TUG: Time Up and Go. (E) Heat map illustrating the various preclinical population characteristics and species in which distinct skeletal muscle clinical endpoints were measured (low values: blue, high values: red). Mass endpoints include measurements both normalized and not normalized to body weight. When forelimb/hindlimb or 4‐limb grip strength was not specified in the study, measurements were considered as four‐limb grip strength. Stimulated strength was measured on either one or more of the individual muscles stated under muscle mass and/or the diaphragm. CKD: chronic kidney disease, D‐gal: D‐galactose, HFD; high‐fat diet, MI: myocardial infarction, NI: no intervention, OA: osteoarthritis, SCI: sciatic nerve injury, WT: wild type.

The age distribution of participants in human studies is shown in Figure 3A. In 49.1% of the studies, the mean age of participants is over 70 years, whereas in 24.5%, the mean age ranges from 60 to 70 years. In the animal studies, 58.7% of mouse experiments and 66.6% of rat experiments involve young animals (0–6 months); 12.7% and 33.3% of mouse and rat experiments, respectively, did not report the age of the animals (Figure 3B). Most human studies (85.2%) enroll individuals of both sexes. However, although human observational studies have a balanced sex ratio, females comprise only 23.9% of participants in human interventional studies (Figure 3C). Similarly, animal experiments are predominantly conducted on male animals, with only 9.1% of mice and 12.4% of rats being female. Only one study includes both male and female mice. In four (6.0%) studies, the sex of the animals is not reported. Studies involving nonrodent animals are conducted on male monkeys, male and female fruit flies and hermaphrodite roundworms (information on frogs not available) (Figure 3C).

In 80.3% of the human studies, the study population consists of nonhospitalized participants. Among these studies, 44.9% select participants based on a specific clinical condition, with Type 2 diabetes and chronic heart failure being the most common (Figure 3D). Of all animal experiments, 43.0% are performed on healthy animals without surgical intervention or muscle wasting induction, whereas the remaining 57.0% involve muscle wasting models, such as denervation or immobilization (Figure 3E). A range of rodent strains are used, most frequently of a C57BL/6 background. Several studies employ genetically modified mouse models—such as peptide or receptor knockouts—including apelin, neuropeptide Y, ghrelin and the ghrelin and Mas receptors. Detailed information on the strain, genotype, and model characteristics for each study is provided in Supporting Information S2.

Of all studies, 56.3% focus on a single muscle wasting component: 38.0% on muscle mass, 12.7% on muscle strength and 5.5% on physical performance. A combination of muscle mass and strength is analysed in 16.6% of studies, whereas 7.9% examine both muscle mass and physical performance. Additionally, 12.7% of studies investigate all three muscle wasting components individually, and 6.3% analyse sarcopenia as a composite variable based on the European Working Group on Sarcopenia in Older People (EWGSOP) or the Asian Working Group for Sarcopenia (AWGS) guidelines. Overall, muscle mass is assessed in 67.4% of studies, muscle strength in 50.0% and physical performance in 26.2%. In human studies, muscle mass is most commonly assessed by ASMM/ASMI (58.6%), strength by handgrip dynamometry (72.1%) and physical performance by gait speed (68.7%) (Figure 3D). Other muscle mass measurements include whole body lean mass (18.9%), fat‐free mass (15.5%) and arm/calf circumference (1.7%). Beyond handgrip strength, muscle strength assessments in humans include knee flexion/extension (20.9%), respiratory muscle strength (2.3%), maximum jumping force (2.3%) and cycling power (2.3%). Additional physical performance metrics include the Short Physical Performance Battery (SPPB, 6.2%), the Timed Up and Go (TUG) test (6.2%), stair climbing (3.1%) and flexibility tests (3.1%). In animal studies, the primary outcomes for muscle mass include whole body lean mass (22.5%) and isolated muscle mass (76.6%), with measurements focusing on specific muscles such as gastrocnemius (25.0%), tibialis anterior (22.5%), soleus (12.9%), extensor digitorum longus (8.8%), plantaris (4.0%) and quadriceps (3.2%). For muscle strength, animals are assessed using voluntary methods (56.1%) such as grip strength (forelimb, hindlimb and four‐limb tests), string hang time and inverted grid tests, as well as involuntary methods (43.9%) like ex vivo and in vivo electrically stimulated force generation. In terms of physical performance, animals are predominantly evaluated by running distance or time (60.7%), with additional metrics including maximum running speed (10.7%), swimming duration (7.1%), pole balance time (3.5%), post‐injury gait analysis (3.5%), walking time in older mice (3.5%) and a decrease in force over time (3.5%) (Figure 3E).

**FIGURE 4:**
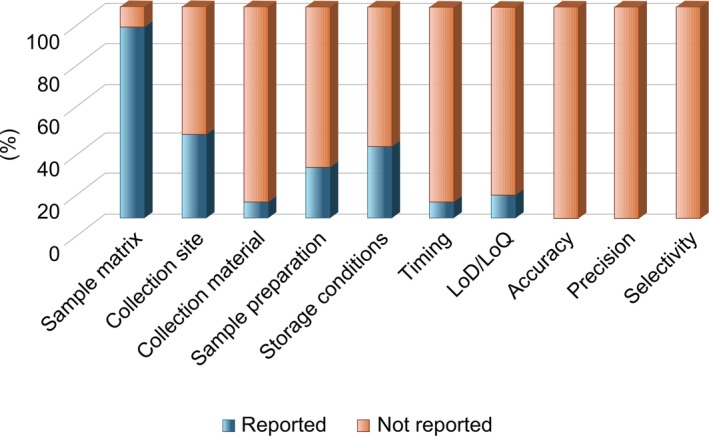
Bar graph showing the proportion of studies in which important pre‐analytical and analytical operating modalities of peptide analysis were reported.

Of the studies measuring peptide concentrations (*n* = 52), 69.8% provided some contextual information regarding the diurnal timing, food intake and physical activity, all of which are well‐known biological factors influencing peptide levels. However, only 56.6% reported all three variables comprehensively. Regarding pre‐analytical factors, 90.3% of studies reported the sample matrix (plasma or serum), 39.6% documented the collection site (peripheral vein), but only 7.5% specified the type of collection materials used (e.g., plastic Eppendorf or EDTA‐coated tubes). Sample preparation details, including centrifugation conditions and solvents used, were provided in 24.5% of studies, and none of the studies specified the duration of sample preparation and indicated whether quantification occurred immediately after preparation. Storage conditions of the biofluids were mentioned in 33.9% of studies, but no studies provided information on the duration of sample storage or the stability of peptides during this period. In terms of analytical characteristics, only 11.5% of studies reported the limit of detection (LOD), and none provided details on selectivity, accuracy or precision (Figure 4).

### 3.2 Peptides Identified

Eighty‐seven chemically distinct peptides were identified, ranging from the shortest, a collagen tripeptide fragment (GPH), to the longest, insulin, containing 51 amino acids (summary per peptide in Table 1; study‐level details in Supporting Information S2). The amino acid sequence is reported in 29.5% of cases, whereas additional structural details including post‐translational modifications or the precise nature of the amino‐ and carboxy‐terminal ends (e.g., amide form) are reported in 12.5% of cases.

**TABLE 1:** Overview and classification by size of the chemically distinct peptides identified throughout the study. Additional information includes the number and type of studies in which they were identified, general biological classification, structural characterization, origin, chemical characteristics and association with muscle wasting endpoints.

Group	Name	N amino acids	N studies	Study type in which found			General biological classification	Structural characterization	Origin	Chemical characteristics				Association with muscle		
				O	HI	A				Hydrophobicity (GRAVY score)	Instability index	Isoelectric point	Boman index (kcal/mol)	Beneficial	Detrimental	Neutral
Small	Gly‐Pro‐Hyp	3	1	0	0	1	Extracellular matrix peptides	Fragment	Mammalian	NA	NA	NA	NA	1	0	1
	SS‐31^a^	4	8	0	0	8	Mitochondrially derived/targeted peptides	De novo peptide	NA	NA	NA	NA	NA	13	0	4
	Cblin^a^	5	2	0	0	2	Cell homeostasis peptides	De novo peptide	NA	NA	NA	NA	NA	2	0	0
	C14 Cblin^a^	5	1	0	0	1	Cell homeostasis peptides	De novo peptide	NA	NA	NA	NA	NA	1	0	0
	BIM‐28131^a^	5	1	0	0	1	Neuropeptides	Natural peptide	Insect	NA	NA	NA	NA	3	0	0
	TCMCB07	9	1	0	0	1	Neuropeptides	De‐novo peptide	NA	NA	NA	NA	NA	1	0	1
	Substituted ACTH (4–9)	6	1	0	0	1	Neuropeptides	Fragment	NA	−4.66	62.67	3.80	2	1	0	0
	C6M	10	1	0	1	0	Extracellular matrix peptides	Fragment	NA	−1.88	20.24	6.00	2.45	0	1	1
	HNK‐1 mimetic peptide	6	1	0	0	1	Other	Natural peptide	Mammalian	−1.66	72.53	9.57	2.16	2	0	0
	[pyr1] Apelin 13^a^	13	1	0	0	1	Neuropeptides	Synthetic derivative	NA	−1.31	72.75	10.84	3	2	0	0
	D‐Apelin 13	13	1	0	0	1	Neuropeptides	Synthetic derivative	NA	−1.31	72.75	12.01	2.9	0	0	1
	IC6	15	1	0	1	0	Extracellular matrix peptides	Fragment	NA	−1.23	46	3.92	3.34	1	0	1
	Cblin‐like‐peptide	5	1	0	0	1	Cell homeostasis peptides	De novo peptide	NA	−0.98	166.78	3.80	1.11	1	0	0
	Melanocyte‐stimulating hormone alpha (a‐MSH)	13	1	1	0	0	GIT and pancreatic peptides	Natural peptide	Mammalian	−0.92	34.02	8.33	2.01	0	1	0
	Proctolin	5	3	0	0	3	GIT and pancreatic peptides	Synthetic derivative	NA	−0.86	31.44	8.75	2.54	1	0	0
	Angiotensin (1–7)^b^	7	5	0	0	5	Neuropeptides	Natural peptide	Mammalian	−0.77	0.54	6.74	2.78	7	0	2
	Substance‐P^b^	11	1	0	0	1	Neuropeptides	Natural peptide	Mammalian	−0.7	47.91	11	1.57	0	1	0
	ARA 284	15	1	0	0	1	Other	Synthetic derivative	NA	−0.65	91.33	4.79	2.23	1	0	0

	FMRF‐a	9	1	0	0	1	Neuropeptides	Natural peptide	Insect	−0.5	34.93	4.37	2.54	1	0	0
	Angiotensin II^b^	7	2	1	0	1	Neuropeptides	Natural peptide	Mammalian	−0.325	25.80	6.74	2.06	0	2	2
	Acein	9	1	0	0	1	Neuropeptides	De novo peptide	NA	−0.31	30.29	9.18	0.73	1	0	0
	YPLP	4	1	0	0	1	Yeast peptide	Natural peptide	Fungal	−0.175	NA	5.52	0.85	1	0	0
	DF‐3	14	1	0	0	1	Myostatin/follistatin‐derived peptides	Fragment	NA	0.01	76.43	5.81	0.8	1	0	0
	ACTH (4–10)^b^	7	1	0	0	1	Neuropeptides	Fragment	NA	0.2	55.14	3.80	1.14	1	0	0
	Growth hormone‐releasing peptide 2 (GHRP‐2)^a^	6	1	0	0	1	GIT and pancreatic peptides	Natural peptide	Mammalian	0.56	−16.72	8.80	0.54	0	0	1
	Rubiscolin‐6	6	1	0	0	1	Neuropeptides	Fragment	Plant	0.66	28.90	5.52	−1	1	0	0
iAM373	7	1	0	0	1	Bacterial peptides	Natural peptide	Bacterial	2.22	8.57	5.24	−1.81	0	3	1
Medium	Acylated ghrelin	28	16	7	3	6	GIT and pancreatic peptides	Natural peptide	Mammalian	NA	NA	11.07	NA	12	2	10
	MZ‐5‐156^a^	29	1	0	0	1	Neuropeptides	Synthetic derivative	NA	NA	NA	NA	NA	1	0	1
	ACTH (1–17)^b^	17	1	0	1	0	Neuropeptides	Fragment	NA	−5.22	50.25	3.50	1.28	1	0	0
	Unacylated ghrelin^b^	28	2	2	0	0	GIT and pancreatic peptides	Natural peptide	Mammalian	−1.67	73.35	11.07	3.62	0	2	0
	MOTS‐c	16	5	2	0	3	Mitochondrially derived/targeted peptides	Natural peptide	Mammalian	−0.93	48.75	10.27	2.63	13	0	0
	MID‐35^a^	16	3	0	0	3	Myostatin/follistatin‐derived peptides	Synthetic derivative	NA	−0.83	97.50	12.01	2.64	4	0	0
	MIPE‐1686^a^	16	1	0	0	1	Myostatin/follistatin‐derived peptides	Synthetic derivative	NA	−0.53	97.50	11.10	1.34	1	0	0
	Peptide‐2 (from mouse myostatin precursor)	24	1	0	0	1	Myostatin/follistatin‐derived peptides	Synthetic derivative	NA	−0.47	122.08	11.57	2.68	2	0	0

	CSP‐7	17	1	0	0	1	Bacterial peptides	Natural peptide	Bacterial	−0.41	108.82	10.90	3.17	0	2	0
	[K15, R16,L27]VIP (1–7)/GRF (8–27)	27	1	0	0	1	GIT and pancreatic peptides	Synthetic derivative	NA	−0.31	26.78	10.43	2.38	0	0	2
	GLP‐1 (7–36)^b^	30	5	5	0	0	GIT and pancreatic peptides	Natural peptide	Mammalian	−0.23	17.69	5.53	1.32	2	5	0
	Obestatin^b^	23	1	1	0	0	GIT and pancreatic peptides	Natural peptide	Mammalian	−0.16	30.38	6.74	0.88	0	2	0
	MBP‐Pro45–70‐His6	26	1	0	0	1	Myostatin/follistatin‐derived peptides	Fragment	NA	−0.12	120	11.01	2.17	3	0	0
	Pep45–65‐NH2	21	1	0	0	1	Myostatin/follistatin‐derived peptides	Fragment	NA	0.15	130	9.50	1.79	3	0	0
	N‐terminal peptide of the PIF receptor (D‐version)^a^	20	1	0	0	1	Other	Fragment	NA	0.29	136.50	5.83	−0.11	1	0	0
	Humanin^b^	24	1	1	0	0	Mitochondrially derived/targeted peptides	Natural peptide	Mammalian	0.341	106.03	9.49	1.27	0	1	0
Endothelin‐1^b^	21	1	0	0	1	Neuropeptides	Natural peptide	Mammalian	0.63	59.44	4.54	0.39	0	3	0
Large	NF‐kappaB inhibitor peptide (NBD peptide)	45	1	0	0	1	Cell homeostasis peptides	De novo peptide	NA	NA	NA	NA	NA	1	0	0
	Apelin^b^	36	3	3	0	1	Neuropeptides	Natural peptide	Mammalian	−1.56	77.96	12.85	3.75	9	1	3
	Peptide YY^b^	36	3	3	0	0	GIT and pancreatic peptides	Natural peptide	Mammalian	−1.20	79.93	8.34	3.21	0	2	1
	Neuropeptide Y^b^	36	1	1	0	0	GIT and pancreatic peptides	Natural peptide	Mammalian	−1.19	63.74	6.76	3	1	0	0
	Crotamine^b^	42	1	0	0	1	Other	Natural peptide	Reptilian	−1.10	82.61	9.51	2.23	0	0	1
	Adropin^b^	43	1	1	0	0	Cell homeostasis peptides	Natural peptide	Mammalian	−1.09	108.88	5.39	2.28	0	1	0

	Ro‐25‐1553 (cyclic)^a^	31	1	0	0	1	GIT and pancreatic peptides	Synthetic derivative	NA	−0.99	20.03	9.78	2.63	4	0	0
	[P11L12L13E22K23A35R36A39R40] Urocortin I	40	1	0	0	1	Neuropeptides	Synthetic derivative	NA	−0.87	97.75	6.35	3.48	1	0	0
	[P11L12L13E22K23A35Q36A39Q40] Urocortin I	40	1	0	0	1	Neuropeptides	Synthetic derivative	NA	−0.82	97.75	4.72	3.01	1	0	0
	Glucose‐dependent insulinotropic polypeptide (GIP)^b^	42	1	1	0	0	GIT and pancreatic peptides	Natural peptide	Mammalian	−0.79	33.32	6.92	1.88	0	1	0
	GHRH‐1,44‐amide^b^	44	1	0	1	0	Neuropeptides	Fragment	NA	−0.79	38.48	10.40	3.03	1	0	1
	[V10P11L12F13E22K23A35R36A39R40] Urocortin I	40	1	0	0	1	Neuropeptides	Synthetic derivative	NA	−0.77	53.10	6.35	3.36	1	0	0
	[P11T12Y13]Sauvagine	40	1	0	0	1	Neuropeptides	Synthetic derivative	NA	−0.72	102.50	6.33	2.23	1	0	0
	[P11T12W13]Sauvagine	40	1	0	0	1	Neuropeptides	Synthetic derivative	NA	−0.71	102.50	6.33	2.17	1	0	0
	[V10P11L12L13E22K23A35Q36A39Q40] Urocortin I	40	1	0	0	1	Neuropeptides	Synthetic derivative	NA	−0.70	105	4.72	2.84	1	0	0
	Exendin‐4^b^	39	2	0	0	2	GIT and pancreatic peptides	Natural peptide	Reptilian	−0.69	64.09	4.70	1.66	6	0	0
	[P11F12Q13]Sauvagine	40	1	0	0	1	Neuropeptides	Synthetic derivative	NA	−0.69	87.58	6.33	2.23	1	0	0
	[P11L12B13]Sauvagine	40	1	0	0	1	Neuropeptides	Synthetic derivative	NA	−0.67	112.25	6.33	2.21	1	0	0
	Midregional pro‐adrenomedullin (MR‐proADM)^b^	48	1	1	0	0	Neuropeptides	Natural peptide	Mammalian	−0.67	87.49	8.59	2.56	0	1	0
	[P11I12Q13]Sauvagine	40	1	0	0	1	Neuropeptides	Synthetic derivative	NA	−0.65	112.25	6.33	2.18	1	0	0
	[P11Q12I13]Sauvagine	40	1	0	0	1	Neuropeptides	Synthetic derivative	NA	−0.65	112.25	6.33	2.18	1	0	0
	Osteocalcin^b^	49	2	2	0	0	Other	Natural peptide	Mammalian	−0.63	67.49	4.40	1.85	0	1	2
	[P11I12Y13]Sauvagine	40	1	0	0	1	Neuropeptides	Synthetic derivative	NA	−0.59	112.25	6.33	2.04	1	0	0
	Alpha‐defensin 26	35	1	0	0	1	Immune system peptides	Natural peptide	Mammalian	−0.54	48.48	9.61	3.05	0	1	0
Sauvagine^b^	40	2	0	0	2	Neuropeptides	Natural peptide	Amphibian	−0.53	122	5.15	2.09	2	0	0

	Brain natriuretic peptide^b^	32	14	13	1	0	Neuropeptides	Natural peptide	Mammalian	−0.50	86.78	10.95	2.43	0	16	2
	[P11I12L13]Sauvagine	40	1	0	0	1	Neuropeptides	Synthetic derivative	NA	−0.47	122	6.33	1.92	1	0	0
	Urocortin I^b^	40	2	0	0	2	Neuropeptides	Natural peptide	Mammalian	−0.44	109.75	5.58	2.85	2	0	0
	Midregional proatrial natriuretic peptide (MR‐proANP)^b^	38	1	1	0	0	Neuropeptides	Natural peptide	Mammalian	−0.37	81.18	11.62	2.63	0	1	0
	GLP‐2^b^	33	2	0	1	1	GIT and pancreatic peptides	Natural peptide	Mammalian	−0.28	6.13	4.17	1.94	2	0	1
	Copeptin^b^	39	1	1	0	0	Neuropeptides	Natural peptide	Mammalian	−0.23	54.82	4.11	1.24	0	0	1
	Alpha‐defensin 5	32	1	0	0	1	Immune system peptides	Natural peptide	Mammalian	−0.11	13.79	8.96	2.6	0	0	1
	Compound B ([E22K23Q24E25K26E27K28Q29]human Urocortin II)	38	1	0	0	1	Neuropeptides	Synthetic derivative	NA	0.02	49.93	8.50	1.51	3	0	0
	C‐peptide^b^	31	14	12	0	2	GIT and pancreatic peptides	Natural peptide	Mammalian	0.03	52.65	3.50	0.28	14	1	3
	ACTH (1–39)^b^	39	1	0	0	1	Neuropeptides	Fragment	NA	0.04	41.01	4.30	1.22	1	0	0
	Compound A ([E22K23Q24E25K26E27K28C29] human Urocortin II)	38	1	0	0	1	Neuropeptides	Synthetic derivative	NA	0.17	52.28	8.18	1.33	4	0	1
	Alpha‐calcitonin gene‐related peptide (a‐CGRP)^b^	37	1	0	1	0	Neuropeptides	Natural peptide	Mammalian	0.21	32.84	9.50	1.08	1	0	0
	Insulin^b^	51	13	11	2	0	GIT and pancreatic peptides	Natural peptide	Mammalian	0.21	NA	5.4	NA	10	3	4
	Abaloparatide (ABL)	34	1	0	0	1	Other	Synthetic derivative	NA	0.45	32.33	10.02	0.61	0	0	1
	Urocortin II^b^	38	2	0	0	2	Neuropeptides	Natural peptide	Mammalian	0.49	37.47	11.40	1.28	6	0	0
MS 9a‐1	35	1	0	0	1	Neuropeptides	Natural peptide	Cnidaria	0.52	14.21	8.92	0.72	1	0	0
Unknown	Bim‐28 125^a^	NA	1	0	0	1	GIT and pancreatic peptides	Synthetic derivative	NA	NA	NA	NA	NA	1	0	0
	Type I collagen carboxyl‐terminal peptide β glypeptide (β‐CTX)	NA	1	1	0	0	Extracellular matrix peptides	Fragment	NA	NA	NA	NA	NA	0	1	0
	Total		176	71	12	94								167	55	50

^a^ Contains non‐human, modified or D amino acids.

^b^ Canonical sequence obtained from Uniprot. A: animal, GIT: gastrointestinal tract, HI: human interventional, N: number, NA: not available, O: observational.

Among these peptides, 62.1% are natural, of which 16.1% are classified as cryptides (hidden fragments of proteins), whereas 37.9% are synthetic. The synthetic peptides include both chemically modified derivatives of natural peptides and de novo sequences. Modified derivatives are created by altering the amino acid sequence of natural peptides and/or adding non–amino acid chemical moieties. Bioactive peptides are sometimes categorized as endogenous (produced by the human body) or exogenous (derived from natural sources or biosynthesis) [19]. However, this distinction is ambiguous because of the discovery of bioactive peptides synthesized by bacteria within the human body and bioactive peptides produced in the human body but also ingested through food. Of the natural peptides, 82.5% are of mammalian origin, whereas the remaining peptides are derived from various sources: reptilian (5.0%), amphibian (2.5%), bacterial (2.5%), fungal (2.5%), cnidarian (2.5%) and insect (2.5%) (Table 1).

The identified peptides can also be classified based on their biological characteristics. The largest group comprises neuropeptides (47.1%), which influence both the central and peripheral nervous systems and facilitate signaling between endocrine and neurological cells. Gastrointestinal and pancreatic peptides represent the second largest group (19.5%). Other peptide groups include myostatin or follistatin derivatives (6.9%), peptides involved in general cell homeostasis (5.7%), extracellular matrix peptides (4.6%), mitochondrially derived or targeted peptides (3.4%), bacterial and yeast peptides (3.4%) and immune peptides (2.2%). The remaining 6.9% of peptides are associated with diverse organ systems not included in any of these groups.

Regarding their effects on muscle, at the reported concentrations, 54.0% of the peptides are associated with beneficial outcomes, 17.2% with detrimental effects and 9.2% are classified as muscle neutral. The remaining 19.5% exhibit mixed effects, with some studies reporting muscle‐beneficial outcomes, whereas others indicate neutral or detrimental effects (Table 1). Supporting Information S3 provides a component‐level summary of muscle outcomes (muscle mass, strength, physical performance or sarcopenia concept) associated with each peptide, whereas study‐level details are presented in Supporting Information S2.

Peptides can also be classified based on size and physicochemical properties, including hydrophobicity (measured by the Grand Average of Hydropathy or GRAVY score), instability index, isoelectric point and Boman index, as detailed in Table 1. Among the 87 peptides, 31.0% are small (3–15 amino acids), 19.5% are medium‐sized (16–30 amino acids), and 47.1% are large (31–51 amino acids). The amino acid length of 2.3% of peptides could not be reliably confirmed. In terms of hydrophobicity, 66.6% of peptides are hydrophilic (GRAVY score < 0), whereas 20.7% are hydrophobic (GRAVY score > 0). The chemical characteristics of 12.6% of peptides containing unnatural amino acids could not be calculated. Furthermore, 25.3% of peptides exhibit an instability index lower than 40, indicating that the majority of peptides are inherently unstable [20]; 48.2% of peptides have an isoelectric point below 7, reflecting a balance between acidic and basic peptides. Finally, the Boman index, which indicates protein‐binding potential, is below 1 for only 13.8% of peptides and below 2 for 35.6% of peptides, suggesting a high protein binding potential for the majority of peptides [21].

### 3.3 Cellular Pathways of Identified Peptides

**FIGURE 5:**
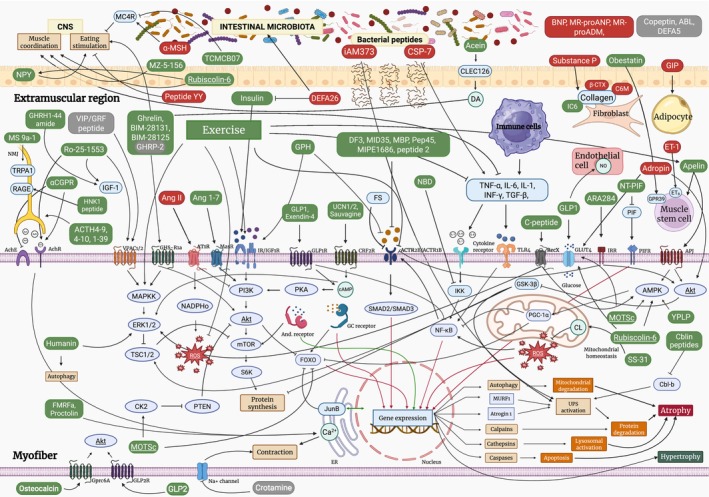
Peptide‐mediated cellular pathways in skeletal muscle. This schematic provides a comprehensive overview of peptide‐regulated signalling pathways in skeletal muscle, summarizing current knowledge from the literature on how peptides influence muscle cell state and fate. The diagram is structured into three major sections: the myofibre, the adjacent extramuscular region, and distant regions, including the central nervous system and gut microbiota, which are highlighted in yellow light. Peptides are colour‐coded based on their overall effect, with green representing peptides associated with muscle hypertrophy, red indicating peptides linked to muscle atrophy, and grey denoting peptides with a neutral effect. Protein mediators are depicted in blue, small‐molecule mediators in light green, general homeostatic processes in light brown and general atrophy‐inducing processes in orange. Arrow‐tipped lines signify stimulation, whereas T‐shaped lines represent inhibition. Gene expression pathways that promote muscle hypertrophy are highlighted in green, whereas those driving muscle atrophy are marked in red. Certain intracellular factors are duplicated for clarity and are underlined to indicate their repeated appearance.

Figure 5 displays an integrated map of musculocellular pathways and extramuscular systems, highlighting the presumed muscle wasting targets and downstream processes influenced by the identified peptides. These insights are derived from mechanistic insights in the articles cited within the studies of this systematic review (snowball approach). Key signalling cascades influencing muscle atrophy and hypertrophy include phosphoinositide 3‐kinase/protein kinase B/mammalian target of rapamycin (PI3K/Akt/mTOR)—either directly or through mitogen‐activated protein kinase/extracellular signal‐regulated kinase/tuberous sclerosis complex 1/2 (MAPK/ERK/TSC1/2)—activin receptor type IIB/mothers against decapentaplegic homologue 2/3 (ACTR2B/SMAD2/3), IκB kinase/nuclear factor kappa‐light‐chain‐enhancer of activated B cells (IKK/NF‐κB) and AMP‐activated protein kinase/peroxisome proliferator‐activated receptor gamma coactivator 1‐alpha (AMPK/PGC1α). These pathways, along with others where peptides are believed to exert their effects, interact with major downstream factors that impact muscle mass. Transcription factors such as FOXO, NF‐κB, SMAD2/3, the glucocorticoid receptor and the protein kinase glycogen synthase kinase 3 beta (GSK‐3β) are crucial downstream regulators that promote the transcription of genes associated with muscle atrophy such as muscle RING‐finger protein‐1 (MuRF‐1) and atrogin‐1. In contrast, transcription factor androgen receptor and transcription cofactor PGC1a drive the expression of genes promoting muscle hypertrophy, whereas ribosomal protein S6 kinase (S6K) enhances muscle hypertrophy by facilitating ribosomal translation as a key mechanism [1, 22, 23, 24]. The map also incorporates exercise, the current ‘gold standard’ for mitigating muscle wasting, with its major downstream pathways [25]. A more detailed textual description of the peptides and their proposed mechanisms of action is provided in Supporting Information S4.

## 4 Discussion

This scoping review identified 87 peptides across 126 studies (period 1981–2024) investigating their association with major clinical components of muscle wasting. Peptides are attractive biological compounds because of their high target affinity and specificity, similar to biologics like therapeutic proteins while also offering the advantages of small molecules. Compared to biologics, peptides have lower immunogenicity and production costs, and unlike small molecules, their larger size, natural human presence, and expanded chemical space—resulting from the diverse side‐chain variations of native amino acids—enhance their target affinity and specificity. Additionally, their structural flexibility enables interaction with broader protein interfaces, making them particularly well‐suited for targeting protein–protein interactions (PPIs). Furthermore, their high biodegradability contributes to greater environmental sustainability compared to small molecules or biologics [26, 27]. Advances in analytical technologies, synthesis and modifications have propelled the use of peptides as biomarkers and therapeutics. Although insulin was the only approved peptide drug up to 1950, subsequent decades saw approvals of simpler hormone mimetics like oxytocin and vasopressin. Recent progress has enabled the development of complex peptides with improved properties such as setmelanotide, a cyclic melanocortin‐4 analogue containing D‐amino acids, and lipid‐conjugated GLP‐1 agonists like semaglutide, liraglutide and dulaglutide [26, 28]. Since 2000, more than 33 novel non‐insulin peptide drugs have been approved, reflecting the growing use of peptide‐based solutions [26, 29, 30].

In muscle wasting diseases, the interest in peptides is driven by the urgent need for biomarkers and therapies for conditions like sarcopenia and cancer cachexia, which currently lack approved treatments in the United States or Europe. Publications on peptides in muscle wasting have grown exponentially over the past 20 years, paralleling the broader interest in peptides. Notably, Japan, the United States and China dominate research output, contributing 23.8%, 20.6% and 13.5% of the 126 studies, respectively. Patent data corroborate these findings, with Japan (24.1%), China (20.7%) and the United States (17.2%) leading in filings for patents targeting skeletal muscle (Espacenet; ‘peptide’ in title and ‘skeletal muscle’ in abstract; *N* = 29). Although China and the United States are among the most populous nations, Japan ranks 12th globally [31]. The disproportionate contribution from Japan may reflect its historical focus on bioactive peptides derived from traditional foods (e.g., fermented products and seafood) and its extensive research into age‐related diseases like cachexia and sarcopenia, aligned with its aging population—the oldest globally.

The identified peptides encompass a diverse range of physicochemical and biological properties. Muscle wasting‐associated peptides vary significantly in size, including small peptides such as Ang (1–7) and SS‐31, medium‐sized peptides like ghrelin and GLP‐1 and larger peptides such as C‐peptide, BNP and insulin. Notably, shorter peptides like SS‐31 and C14 Cblin often exhibit superior tissue and cellular penetration and tend to have lower immunogenicity compared to their longer counterparts [32, 33]. Conversely, longer peptides, such as insulin, can facilitate more complex and highly specific interactions with cellular targets [34]. Beyond size, the peptides also demonstrate variability in hydrophobicity and protein‐binding potential within each size category. Hydrophilic peptides like Ang (1–7) (small), ghrelin (medium) and apelin (large) exhibit high protein‐binding potential. In contrast, hydrophobic peptides such as iAM373 (small), the N‐terminal peptide of the PIF receptor (medium) and C‐peptide (large) show low protein binding potential, with no clearly identified receptors [35, 36, 37]. Although natural peptides with high protein‐binding potential may serve as versatile multi‐target agents in vivo, therapeutic applications often prioritize low protein‐binding peptides. Such peptides are more desirable because of their high specificity and reduced likelihood of off‐target interactions, making them ideal for precision therapeutics [38]. Rapid degradation is also a limiting factor in peptide therapeutics, with chemical modifications being commonly employed to achieve the desired stability of the therapeutic peptide, exemplified by insulin glargine and semaglutide [39].

Nearly two‐thirds (62.1%) of the investigated peptides exert their effects through just four primary cellular pathways: PI3K/Akt/mTOR (directly or via MAPK/ERK/TSC1/2), ACTR2B/SMAD, IKK/NF‐kB and AMPK/PGC1α. This distribution follows a Pareto‐like pattern, where a limited number of pathways account for the majority of observed effects. The PI3K/Akt/mTOR pathway is a key regulator of muscle growth and metabolism. PI3K phosphorylates the serine/threonine kinase Akt, which in turn activates the mTORC1 complex—comprising mTOR and scaffold proteins like Raptor [40]. Akt and mTORC1 promote protein synthesis primarily by inhibiting FOXO and stimulating S6K [40, 41]. The TSC1/TSC2 complex, regulated by MAPK–ERK, acts as a negative regulator of mTOR by inactivating Rheb, an activator of mTOR [42]. Peptides influence this pathway via diverse receptors, including IR, IGF‐1R, MasR, AT1R, VPAC1/2R, GHS‐R1a, APJ, GLP‐2R, GPCR61 and IRR. Targeting receptors with high skeletal muscle expression could help minimize off‐target effects. For example, the apelin receptor (APJ) is a promising candidate, as it is relatively more expressed in skeletal muscle than in other tissues, such as the respiratory and renal systems [43]. In contrast, targeting IGF‐1R with IGF‐1‐derived peptides, such as its C‐terminal E‐sequences, is less muscle‐specific and carries a risk of adverse effects, including myalgia, oedema, hypoglycaemia, seizures, jaw pain, headaches, altered liver function and increased liver and kidney mass, as reported with recombinant human IGF‐I administration [44]. Another receptor of interest is CRF2R, an indirect activator of the PI3K/Akt/mTOR pathway via cAMP, which is stimulated by urocortin peptides. Notably, CRF2R shows relatively high expression in skeletal muscle, in addition to the brain. cAMP has recently been identified as a causal signalling mediator between blood metabolites and sarcopenia [45]. Also relevant for future research are cellular communication network (CCN) family proteins, such as WISP‐1, an extracellular matrix protein that promotes muscle stem cell commitment by activating the Akt pathway [46].

The ACTR2B/SMAD2/3 pathway mediates the effects of myostatin, follistatin, and their derivatives on muscle. Myostatin negatively regulates skeletal muscle growth by sequentially binding to activin type II (mainly ActRIIB) and type I receptors (mainly ActRIB), activating the Smad2/3 transcription pathway. Smad2/3 promotes muscle atrophy, in part, by increasing the transcription of the ubiquitin ligase MuRF‐1, partly through enhanced FoxO3 binding to the MuRF‐1 gene [47]. Although targeting this pathway holds promise for combating muscle wasting, many clinical trials have failed because of the low tissue specificity of ACTR2B and SMAD2/3 at both mRNA and protein levels, resulting in adverse events and off‐target effects [43, 48]. Advancements such as muscle‐targeted delivery systems, including exosomes—naturally produced by and affecting human muscle cells—could improve the specificity and safety of therapies targeting this pathway [49, 50]. Additionally, clinical trials of myostatin inhibitors have shown significant increases in muscle mass but inconsistent improvements in muscle strength or physical performance. One possible explanation for this mass–strength discrepancy is that whereas muscle fibre size increases, the number of myonuclei does not, leading to fibre demands exceeding myonuclear capacity [51]. Maintaining an appropriate myonuclear domain—where each nucleus regulates a feasible portion of the muscle fibre—may be crucial to buffering gene expression variability and ensuring functional muscle adaptation [52].

NF‐κB is a key regulator of inflammation, apoptosis, and muscle atrophy. In its inactive state, NF‐κB is sequestered in the cytoplasm by IκB proteins. IκB undergoes phosphorylation by IκB kinase (IKK) and subsequent degradation via the ubiquitin–proteasome system, freeing NF‐κB to translocate into the nucleus, where it activates target gene expression [53]. Elevated levels of pro‐inflammatory cytokines like TNFα, IL‐6, IL‐1 and IFNγ, observed in catabolic conditions, drive muscle wasting through increased NF‐κB activity [54]. Additionally, activation of muscular Toll‐like receptor 9 or the cGAS‐STING pathway by mitochondrial or nucleic DNA released upon cellular damage further stimulates NF‐κB signalling and muscle degradation [55, 56]. In this context, the cGAS‐STING inhibitory peptide ISD017, which has shown anti‐inflammatory effects in preclinical lupus models, warrants investigation for its potential to mitigate muscle wasting [56]. More broadly, peptides that modulate inflammatory pathways could not only improve muscle outcomes but also benefit systemic inflammatory conditions, including cardiovascular and autoimmune diseases, as well as aging‐related chronic inflammation, commonly referred to as ‘inflammaging’ [57, 58]. Of particular interest is the recently discovered sarcopenia‐preventing effect of gamma‐aminobutyric acid (GABA), an amino acid–derived neurotransmitter. In a preclinical study, GABA supplementation for 7 weeks led to a 40%–70% increase in muscle strength in aged mice, likely through inflammation reduction and Akt/mTOR pathway activation [59]. Notably, peptides incorporating GABA as an amino acid derivative have already been synthesized in nonmuscle contexts, highlighting potential avenues for future therapeutic development [60]. Although pro‐inflammatory cytokines beyond acute exercise are typically associated with muscle wasting, recent findings suggest that cytokines like IL‐13 can counteract this process by stimulating mitochondrial activity in muscle cells [61]. IL‐13‐inducing peptides are currently being identified through in silico approaches, also offering promising potential for treating muscle wasting diseases [62].

AMP‐activated protein kinase (AMPK) is activated by AMP, serving as a key sensor of cellular energy status. Upon activation, AMPK phosphorylates PGC‐1α, a master regulator of mitochondrial biogenesis. PGC‐1α influences energy metabolism by activating transcription factors such as PPARy and Yin‐Yang 1 (YY1) while inhibiting NF‐κB, thereby linking energy regulation to inflammation [63, 64]. Notably, YY1 is also a target of mTOR, highlighting the crosstalk between the AMPK/PGC‐1α and PI3K/Akt/mTOR pathways [65]. This interplay between energy and growth regulation is further reflected in mTOR's role as a critical regulator of mitophagy, the selective autophagic degradation of damaged or aging mitochondria [66]. Additionally, recent research has identified extended synaptotagmin 1 (E‐Syt1) as a key regulator of PGC‐1α in muscle wasting, although its interactions with peptides remain to be explored [67]. These muscular mitochondrial pathways are critical for maintaining whole body energy homeostasis [68]. Accordingly, peptides such as MOTS‐c and YPLP hold promise for counteracting muscle wasting by enhancing muscular mitochondrial function and optimizing whole body energy metabolism [14, 69, 70, 71, 72].

In this scoping review, we observed that significant gaps in study design and reporting are prevalent, hindering clinical translation. For study design, male overrepresentation and clinical outcome variability are notable issues. Human observational studies show relatively balanced male and female participation; however, in human interventional studies, only 23.9% of participants are female, and in rodent studies, females constitute just 12.4% of rats and 9.1% of mice. These figures are within the typical range for other research areas—between 20% and 50% for female involvement in human clinical trials and about 20% in animal research—but they are at the lower end of these ranges [73, 74]. The documented sex differences in skeletal muscle gene expression, kinetics, fibre‐type composition and aging mechanisms highlight the importance of addressing this disparity [75, 76]. Fortunately, there is growing awareness and an increasing trend in female representation in research [77, 78]. For the clinical outcomes, in human studies, ASMI, handgrip strength and gait speed are the most commonly used muscle‐related endpoints, aligning with recommendations from international sarcopenia working groups [79, 80]. However, in animal studies, from the isolated muscles weighted to assess muscle mass, only one third evaluate the M. gastrocnemius. It is critical to prioritize gastrocnemius in aging‐related muscle wasting rodent studies, given that this muscle is most important for locomotion and also most affected by sarcopenia in wild‐type mice [81]. Furthermore, muscle mass outcomes are frequently not normalized to anthropometric measures in both human and animal studies, whereas evidence suggests more accurate results when normalized to height, weight, or both [82]. For muscle strength in animals, a distinction can be made between voluntary methods such as grip strength and involuntary methods such as ex vivo muscle contraction. Involuntary methods more accurately and precisely measure muscle strength but are invasive and require specialized equipment and training [83]. Remarkably, cancer cachexia populations are underrepresented in human peptide–muscle wasting studies, and only 11.1% of animal experiments are conducted on cancer models. This might be due to the limited focus on skeletal muscle in cachexia clinical trials, where only 24% of clinical outcomes involve muscle measurements, whereas the rest focus on body weight [84]. Current guidelines for cancer cachexia emphasize the use of body weight alongside radiological modalities to enhance clinically relevant outcomes [82]. Additionally, only 17.4% of human studies and 18.3% of animal studies assess muscle mass and either muscle strength and/or physical performance, collectively referred to as muscle function. The relative importance of muscle mass versus function has evolved and continues to be debated. Both elements are likely complementary and differ in their sensitivity to interventions depending on the stage of muscle wasting in the subject. Ramage and Skipworth have proposed an interesting non‐linear relationship between muscle mass and muscle function in cancer cachexia over a clinical timeline, which could also be applicable to other muscle wasting conditions [85]. Therefore, we advocate for future muscle wasting studies to include assessments of both muscle mass and muscle function. The specific choice of measurement should ideally be based on the intervention's intended effects, for example, SPPB when balance and strength are expected to change.

We also observed significant gaps in reporting the exact chemical peptide structure as well as clinical, pre‐analytical and analytical parameters in the reviewed studies. Precise information on the chemical structure, including modifications and the precise nature of the amino‐ and carboxy‐terminal ends, is crucial, as these factors significantly influence peptide function. For example, acylated (active) ghrelin promotes food intake, whereas deacylated ghrelin reduces food intake [86]. Clinical conditions of blood collection such as whether subjects are fed or fasted, the diurnal timing of collection and exercise levels before collection are known to significantly impact peptide concentrations—resulting in variations of over 350% [87, 88, 89, 90, 91, 92]. Pre‐analytical variables, such as the type of collection tube used and the timing between blood collection, storage and sample preparation, are also critical; for example, certain peptides exhibit a tenfold increase in half‐life when collected in P100 tubes compared to EDTA tubes, attributed to the protease inhibition properties of P100 tubes [93]. Additionally, a delayed sample preparation process can result in a loss of over 60% in peptide concentration compared to a rapid preparation process in human biofluids [94]. Furthermore, none of the studies report stability data for these steps. In terms of traditional analytical parameters like LOD, selectivity, accuracy and precision, the reporting rates are low. Only 11.5% of studies report the LOD, and none provide details on selectivity, accuracy or precision.

Despite their biological effects on muscle, none of the 87 peptides reviewed—whether in their natural or modified forms—has been approved specifically for muscle wasting diseases, highlighting a gap in clinical translation. However, some peptides or their analogues are approved for other medical indications, including insulin (Type 1/2 diabetes), angiotensin II (critical hypotension), GHRH (GH deficiency), abaloparatide (osteoporosis), the GHRH derivative goserelin (prostate and breast cancer, endometriosis and uterine bleeding), the ghrelin analogue relamorelin (gastroparesis), the α‐CGRP antagonist rimegepant (migraine) and ARA‐284 erythropoietin analogues (anaemia) [95, 96, 97]. Given their established safety profiles, repurposing these peptides for muscle wasting disorders presents a cost‐effective therapeutic strategy. Of particular recent interest are GLP‐1 receptor agonists such as semaglutide and liraglutide, widely used for Type 2 diabetes and weight management [98]. Although these drugs effectively reduce body weight, a significant portion of the lost weight (25%–39%) is lean mass, following a similar linear relationship between lean mass and total body weight loss as seen with caloric restriction. Muscle strength is often preserved, indicating a higher quality of the remaining lean mass [99, 100, 101, 102]. However, unlike caloric restriction, GLP‐1 therapy may pose a higher risk of rapid weight loss–regain cycles upon treatment discontinuation and resumption. Such fluctuations can exacerbate muscle loss, decrease strength and increase the risk of sarcopenic obesity [103, 104]. The full extent of GLP‐1‐induced muscle effects remains unclear, partly because of the lack of FDA and EMA requirements to assess both muscle mass and function in weight loss drug trials [101, 105]. However, ongoing research is exploring strategies to mitigate lean mass loss from GLP‐1 therapy, including adjunctive approaches such as protein intake, exercise, bimagrumab and enobosarm [99, 104].

Several peptides are not yet approved but are under clinical investigation for nonmuscle indications, including apelin (antihypertensive) and the apelin–receptor agonist Elabela (cardioprotective and renoprotective), urocortin I (cardioprotective) and MOTS‐c (metabolic diseases) [106]. Currently, TMCMB07, myostatin, Cblin derivatives, CSP‐7 and iAM373 are the only peptides exclusively studied for their effects on skeletal muscle [107, 108, 109, 110, 111, 112, 113, 114, 115]. Notably, iAM373, a bacterial peptide, has been shown to reduce metabolic activity in muscle cells and impair muscle function in vivo. Although this identifies iAM373 as a negative regulator of muscle metabolism, its potential benefits under specific conditions remain unexplored. Growing interest in hibernation pathways, where animals downregulate muscle metabolism to prevent muscle loss during prolonged inactivity, suggests that targeted metabolic suppression could have therapeutic applications [116].

The growing complexity and diversity of peptide functions highlight the need for more systematic discovery strategies. In this context, machine learning (ML)—a branch of artificial intelligence focused on detecting patterns in complex datasets for predictive modeling [117, 118]—offers a promising avenue. ML has already been successfully applied to identify bioactive peptides with anticancer, antiviral, and antibacterial properties [119], as well as to predict key pharmacokinetic and pharmacodynamic properties, such as cell permeability, blood–brain barrier penetration, and receptor binding [120, 121, 122, 123, 124]. These models can be trained on sequence‐ or structure‐based peptide data and often rely on correlation and multivariate analyses to identify the most informative molecular descriptors for each peptide class [125]. In the context of peptides active against skeletal muscle loss, future research may benefit from three ML‐driven strategies: (i) exploring the peptide chemical space identified in this review to establish molecular filters for virtual screening of new candidates; (ii) applying generative models—such as generative adversarial networks (GANs) [126] and variational autoencoders (VAEs) [127]—to design novel peptides based on learned structural patterns; and (iii) developing large language model (LLM)–based AI agents to support automated extraction and synthesis of literature on muscle wasting [128]. Together, these approaches offer a powerful framework to support the discovery of novel peptide‐based interventions targeting muscle wasting.

## 5 Strengths and Limitations

This scoping review provides the first comprehensive overview of peptides associated with muscle wasting, offering researchers and clinicians a global perspective on the current state‐of‐the‐art in this field. A key strength of this review is its exploration of the mechanisms by which peptides—in animal and human studies—influence muscle wasting, identifying the specific pathways being targeted. Furthermore, the review highlights critical design and reporting gaps in existing studies, offering valuable guidance to improve the quality of future research.

However, some limitations should be acknowledged. First, we did not conduct a meta‐analysis or quantify effect sizes because of the substantial heterogeneity in study populations, measurement tools and outcomes, which exceeds a scoping review. Second, our focus was limited to objective muscle wasting variables, excluding quality‐of‐life outcomes—an important clinical measure in muscle wasting research that should be included in future studies. Third, we excluded in vitro studies to concentrate on animal and human research. However, in line with the 3R principles (Replacement, Reduction and Refinement), emerging 3D in vitro muscle wasting models, such as organoids, are expected to play an increasingly important role in mechanistic research and may inform subsequent (animal and) human studies.

## 6 Conclusion

The 87 identified peptides exhibit diverse physicochemical properties, including hydrophobicity and protein‐binding potential, which can be altered through synthetic modifications. Notably, nearly two‐thirds of the peptides act through four primary cellular pathways—PI3K/Akt/mTOR (directly or via MAPK/ERK/TSC1/2), ACTR2B/SMAD, IKK/NF‐kB and AMPK/PGC1α—reflecting a Pareto‐like distribution.

This scoping review highlights pervasive gaps in study design and reporting that impede clinical translation. Future research should address these gaps by ensuring better female representation and assessing both muscle mass and function, with appropriate measurement methods. Reproducibility requires complete reporting of peptide chemical structures, including modifications, as well as comprehensive documentation of clinical, pre‐analytical and analytical parameters.

Finally, future research should incorporate ML approaches to screen, design and automatically identify novel bioactive peptides with therapeutic potential for muscle wasting diseases from the scientific literature.

## Conflicts of Interest

The authors declare no conflicts of interest.

## Supporting information


**Data S1:** Details of the review methods.

**Data S2:** Extracted data from the 126 selected articles.

**Data S3:** Component‐level summary of muscle outcomes (muscle mass, strength, physical performance or composite sarcopenia) associated with each peptide.

**Data S4:** Presumed muscle wasting targets and downstream processes of the peptides.
