# Supplementary material for: Role of Peptides in Skeletal Muscle Wasting: A Scoping Review
Source: J Cachexia Sarcopenia Muscle. 2025 Nov 13;16(6):e70109. doi: 10.1002/jcsm.70109 (PMC12613835; doi:10.1002/jcsm.70109)
Supplement: Supplementary file 1 — Data S1: Details of the review methods. [file JCSM-16-e70109-s003.docx]

**Supporting File S1**

**Research question**

In the current scoping review, our primary objective is to gain a deeper understanding of the role of peptides in muscle wasting, with the following key questions:

1. Which peptides are directly linked to alterations in muscle mass, muscle strength, and/or physical performance in animal or human models?
2. What are the muscle-related cellular effects of these peptides ?
3. What knowledge gaps and quality deficiencies exist, and how can they be effectively addressed in future studies?

Through a comprehensive analysis of the available literature, we aim to shed light on these critical questions.

**Data sources and search strategy**

A systematic literature search for relevant studies was carried out using Embase, Pubmed and Web of Science, from the earliest record of each database up to October 2024. Search entries included a combination of three main aspects: 1) keywords related to peptides, AND 2) keywords related to human participants and preclinical *in vivo* muscle wasting models, AND 3) keywords related to the three main clinical muscle wasting-components, namely muscle mass, muscle strength or physical performance. We limited our search to original articles or letters written in English, including preprints, while excluding reviews, meta-analyses, editorials, and case reports. Additionally, articles with titles mentioning genetic muscle diseases or inflammatory myopathies, as well as those with smooth or cardiac muscle in their title, were excluded from our search. The complete search query applied can be found below.

**Study eligibility criteria**

We included all original studies involving humans or animals, without restrictions based on sex, age or animal model. To capture a broad spectrum of evidence, both observational and interventional studies were included. Eligible studies had to directly assess the association between a specific peptide and at least one of the three key clinical components of muscle wasting: muscle mass, muscle strength, or physical performance. Peptides were defined as amino acid chains consisting of 3 to 51 residues, while compounds with more than 51 residues—such as the myokine irisin (112 amino acids)—were classified as proteins and excluded from this review. Only peptides detected in, or administered to, the systemic circulation or muscle tissue were considered. Studies involving peptide mixtures such as protein/peptide hydrolysates (e.g. sea cucumber peptides affecting muscle mitochondrial function^1^) or those assessing indirect peptide-muscle associations were excluded. Peptides investigated solely in *in vitro* studies without *in vivo* validation were also beyond the scope of this review. Additionally, animal studies related to the food industry and research focusing on congenital or inherited muscle diseases, inflammatory myopathies, or neurodegenerative diseases (e.g., Parkinson’s disease) were not included.

**Screening and article selection**

Two independent reviewers conducted the article screening process using Rayyan software. In the first stage, articles were excluded based on their titles. In the second stage, selection was made by evaluating the abstracts, and in the final stage, the reviewers assessed the full text of the remaining articles. Any disagreements during the process were resolved through discussion, with a third reviewer consulted if consensus could not be reached. The entire screening process was documented using a PRISMA flow diagram.

**Data extraction**

Two reviewers independently extracted all necessary information, resolving any disagreements through discussion with a third reviewer. The extracted data was systematically organized into tables, which included the following information: first author surname, country, year, study design, population and peptide characteristics, intervention details (if applicable), exposure and outcome (including time, measurement conditions, and methods), covariates, and a description of the results along with the direction of association. No assumptions were made regarding missing or unclear data.

**Physicochemical properties of identified peptides**

Hydrophobicity, measured by Grand Average of Hydropathy (GRAVY), and Boman index were calculated using University of Nebraska Medical Center ADP calculator^2^. The Boman Index measures a peptide's potential to interact with other proteins, which is important in assessing its binding capacity to receptors and enzymes. Isoelectric point and instability index were calculated using ProtPram^3^. The instability index is a computational prediction of the peptide’s *in vitro* and *in vivo* stability, based on amino acid composition. The properties of peptides containing amino acids other than the 22 proteinogenic amino acids were not calculated.

**Cellular pathways of identified peptides**

The presumed cellular pathways of the identified peptides were synthesized narratively by integrating data from mechanistic studies on peptide-muscle interactions. Thereto, a snowball approach was applied, where mechanistic references from selected articles in the review were used to create an integrated pathway model highlighting presumed targets and downstream processes associated with the identified peptides.

**References**

1. Wang Q, Shi J, Zhong H, et al. High-degree hydrolysis sea cucumber peptides improve exercise performance and exert antifatigue effect via activating the NRF2 and AMPK signaling pathways in mice. *J Funct Foods*. 2021;86:104677. doi:10.1016/J.JFF.2021.104677

2. Wang G. The antimicrobial peptide database is 20 years old: Recent developments and future directions. *Protein Science*. 2023;32(10). doi:10.1002/pro.4778

3. Garg VK, Avashthi H, Tiwari A, et al. MFPPI – Multi FASTA ProtParam Interface. *Bioinformation*. 2016;12(2):74-77. doi:10.6026/97320630012074

**Complete search query**

1. **EMBASE** [Last searched August 6, 2023]

**(Title, Abstract or Author keywords)**

('peptide' OR 'hormonal peptide' OR 'polypeptide*' OR 'oligopeptide*' OR 'peptide*' OR 'peptide* chain*' OR 'hormonal* peptide*' OR 'hormone* peptide*' OR 'hormone*, peptide*' OR 'peptide* hormone*' OR 'peptide*, hormonal*' OR 'polypeptide* hormone*' OR 'chain, polypeptide*' OR 'peptide*, poly' OR 'polypeptide* chain*' OR 'peptide*, oligo' OR 'bioactive peptide*' OR 'therapeutic* peptide*' OR 'bacterial* peptide*' OR 'exogenous peptide*' OR 'endogenous peptide*' OR 'natural peptide*')

**AND** **(Title, Abstract or Author keywords)**

('human' OR 'trial' OR 'RCT' OR 'first in human study' OR 'patient*' OR 'child*' OR 'animal*' OR 'animal experiment*' OR 'rodent' OR 'murine model' OR 'rat*' OR 'mouse' OR 'mouse model' OR 'rat model' OR 'primate' OR 'Caenorhabditis elegans' OR 'Drosophila' OR 'zebra fish' OR 'human*' OR 'Homo sapiens' OR 'human being*' OR 'human body' OR 'human race*' OR 'human subject*' OR 'man' OR 'first-in-human study' OR 'first-in-man study' OR 'sufferer*' OR 'participant*' OR 'men' OR 'man' OR 'woman' OR 'women' OR 'rodent*' OR 'murin* model*' OR 'Rattus' OR 'mice' OR 'Mus' OR 'newborn mice' OR 'animal population group*' OR 'Animalia' OR 'Metazoa' OR 'metazoan*' OR 'metazoon' OR 'animal* experimentation' OR 'animal* physical conditioning' OR 'animal studies' OR 'animal study' OR 'animal trial*' OR 'experiment*, animal' OR 'physical conditioning, animal' OR 'primate*' OR 'C elegans' OR 'C. elegans' OR 'nematode' OR 'Rhabditis elegans' OR 'roundworm' OR 'drosophile' OR 'Drosophilia' OR 'fruit fly' OR 'Brachydanio rerio' OR 'Cyprinus rerio' OR 'Danio frankei' OR 'Danio rerio' OR 'zebra danio' OR 'zebrafish' OR 'zebra fish')

**AND** **(Title, Abstract or Author keywords)**

('sarcopenia' OR 'muscle function' OR 'muscle strength' OR 'muscle mass' OR 'grip strength' OR 'gait speed' OR 'walking speed' OR 'physical function*' OR 'physical functioning' OR 'physical performance*' OR 'cachexia' OR 'muscle atrophy' OR ‘sarcopenic’ OR 'muscle function*' OR 'function*, muscle' OR 'muscle performance*' OR 'muscular effort*' OR 'muscular function*' OR 'muscular performance*' OR 'muscle work' OR 'muscular work' OR 'performance*, muscle' OR 'work, muscle' OR 'dynamic strength, muscle' OR 'dynamic strength, muscular' OR 'force, muscle' OR 'muscle dynamic strength' OR 'muscle force' OR 'muscle force velocity relationship' OR 'muscle power' OR 'muscular dynamic strength' OR 'muscular force' OR 'muscular power' OR 'strength, muscle' OR 'muscular strength' OR 'muscle volume' OR 'muscle weight' OR 'weight, muscle' OR 'grasp force*' OR 'grasp strength' OR 'grasping strength' OR 'gripping force' OR 'gripping power' OR 'handgrip strength' OR 'abilit*, physical' OR 'assessment*, physical performance*' OR 'evaluation*, physical performance*' OR 'performance*, physical' OR 'physical performance* assessment*' OR 'physical performance* evaluation*' OR 'physical performancy' OR 'cachectic state' OR 'cancer cachexia' OR 'cachexia' OR 'cachectic' OR 'amyotrophia' OR 'amyotrophy' OR 'atrophic muscular disorders' OR 'atrophy type 2' OR 'atrophy, muscle' OR 'degeneration, muscle' OR 'muscle atrophia' OR 'muscle cell degeneration' OR 'muscle degeneration' OR 'muscle fiber atrophy' OR 'muscle fiber degeneration' OR 'muscle recession' OR 'muscle wasting' OR 'muscular atrophy' OR 'muscular degeneration' OR 'muscular disorders, atrophic' OR 'myoatrophy' OR 'myodegeneration' OR 'myofibrillar degeneration' OR 'myophagism')

**AND (Language of article)**

'english'

**AND (Publication type)**

('Article' OR 'Letter' OR 'Article in Press' OR 'preprint')

**NOT (Publication type)**

('review' OR 'meta-analysis' OR 'case report' OR 'case-report' OR 'editorial')

**NOT (Title)**

('dystrophia' OR 'dystrophic syndrome' OR 'dystrophy' OR 'congenital atonic sclerotic muscular dystrophy' OR 'congenital muscle dystrophy' OR 'congenital muscular dystrophy' OR 'dystrophy, muscle' OR 'muscle dystrophia' OR 'muscle dystrophy' OR 'muscular dystrophia' OR 'muscular dystrophies' OR 'muscular dystrophy, congenital' OR 'myodystrophia' OR 'myodystrophy' OR 'muscular dystrophy' OR 'neurogenic muscle disease' OR 'neurogenic myopathy' OR 'neuromuscular diseases' OR 'neuromuscular disorder' OR 'neuromuscular dysfunction' OR 'neuromuscular syndrome' OR 'neuromyopathy' OR 'neuromuscular disease' OR 'myasthenia' OR 'ALS' OR 'amyotrophic lateral sclerosis' OR 'allergic myositis' OR 'idiopathic inflammatory myopathy' OR 'muscle infection' OR 'myositis, allergic' OR 'neuromyositis' OR 'myositis' OR 'Spinal muscular atrophy' OR 'SMA')

**NOT (Title)**

'cardiomyopathy' OR 'smooth muscle' OR 'systolic' OR 'diastolic'

**NOT (Title)**

'in vitro' OR 'in-vitro' OR 'C2C12'

(number of references: 1115)

1. **PubMed** [Last searched August 6, 2023]

**Query: (Title/Abstract)** (peptide OR “hormonal peptide” OR polypeptide* OR oligopeptide* OR peptide* OR “peptide chain*” OR “hormonal peptide*” OR “hormone peptide*” OR “hormone, peptide*” OR “peptide hormone*” OR “peptide, hormonal*” OR “polypeptide hormone*” OR “chain, polypeptide*” OR “peptide, poly” OR “polypeptide chain*” OR “peptide, oligo” OR “bioactive peptide*” OR “therapeutic peptide*” OR “therapeutical peptide*” OR “bacterial peptide*” OR “exogenous peptide*” OR “endogenous peptide*” OR “natural peptide*”)

**AND (Text Word)** (human OR trial OR RCT OR “first in human study” OR patient* OR child* OR animal* OR “animal experiment*” OR rodent OR “murine model” OR “rat” OR “rats” OR “mouse” OR “mouse model” OR “rat model” OR primate OR “Caenorhabditis elegans” OR Drosophila OR “zebra fish” OR human* OR “Homo sapiens” OR “human being*” OR “human body” OR “human race*” OR “human subject*” OR man OR “first-in-human study” OR “first-in-man study” OR sufferer* OR participant* OR men OR man OR woman OR women OR rodent* OR “murin model*” OR Rattus OR mice OR Mus OR “newborn mice” OR “animal population group*” OR Animalia OR Metazoa OR metazoan* OR metazoon OR “animal experimentation” OR “animal physical conditioning” OR “animal studies” OR “animal study” OR “animal trial*” OR “experiment, animal” OR “physical conditioning, animal” OR primate* OR “C elegans” OR “C. elegans” OR nematode OR “Rhabditis elegans” OR roundworm OR drosophile OR Drosophilia OR “fruit fly” OR “Brachydanio rerio” OR “Cyprinus rerio” OR “Danio frankei” OR “Danio rerio” OR “zebra danio” OR zebrafish OR “zebra fish”)

**AND** **(Title/Abstract)** (sarcopenia OR “muscle function” OR “muscle strength” OR “muscle mass” OR “grip strength” OR “gait speed” OR “walking speed” OR “physical function*” OR “physical functioning” OR “physical performance*” OR cachexia OR “muscle atrophy” OR sarcopenic OR “muscle function*” OR “function, muscle” OR “muscle performance*” OR “muscular effort*” OR “muscular function*” OR “muscular performance*” OR “muscle work” OR “muscular work” OR “performance, muscle” OR “work, muscle” OR “dynamic strength, muscle” OR “dynamic strength, muscular” OR “force, muscle” OR “muscle dynamic strength” OR “muscle force” OR “muscle force velocity relationship” OR “muscle power” OR “muscular dynamic strength” OR “muscular force” OR “muscular power” OR “strength, muscle” OR “muscular strength” OR “muscle volume” OR “muscle weight” OR “weight, muscle” OR “grasp force*” OR “grasp strength” OR “grasping strength” OR “gripping force” OR “gripping power” OR “handgrip strength” OR “ability, physical” OR “assessment, physical performance*” OR “evaluation, physical performance*” OR “performance, physical” OR “physical performance assessment*” OR “physical performance evaluation*” OR “physical performancy” OR “cachectic state” OR “cancer cachexia” OR cachexia OR cachectic OR amyotrophia OR amyotrophy OR “atrophic muscular disorders” OR “atrophy type 2” OR “atrophy, muscle” OR “degeneration, muscle” OR “muscle atrophia” OR “muscle cell degeneration” OR “muscle degeneration” OR “muscle fiber atrophy” OR “muscle fiber degeneration” OR “muscle recession” OR “muscle wasting” OR “muscular atrophy” OR “muscular degeneration” OR “muscular disorders, atrophic” OR myoatrophy OR myodegeneration OR “myofibrillar degeneration” OR myophagism)

**NOT (Title)** (dystrophia OR “dystrophic syndrome” OR dystrophy OR “congenital atonic sclerotic muscular dystrophy” OR “congenital muscle dystrophy” OR “congenital muscular dystrophy” OR “dystrophy, muscle” OR “muscle dystrophia” OR “muscle dystrophy” OR “muscular dystrophia” OR “muscular dystrophies” OR “muscular dystrophy, congenital” OR myodystrophia OR myodystrophy OR “muscular dystrophy” OR “neurogenic muscle disease” OR “neurogenic myopathy” OR “neuromuscular diseases” OR “neuromuscular disorder” OR “neuromuscular dysfunction” OR “neuromuscular syndrome” OR neuromyopathy OR “neuromuscular disease” OR myasthenia OR ALS OR “amyotrophic lateral sclerosis” OR “allergic myositis” OR “idiopathic inflammatory myopathy” OR “muscle infection” OR “myositis, allergic” OR neuromyositis OR myositis OR “Spinal muscular atrophy” OR SMA)

**AND (Language)**

“English”

**AND (Publication type)**

(Journal Article OR Letter OR preprint)

**NOT (Publication type)**

(Review OR Systematic Review OR Meta-analysis OR Case Reports OR Editorial)

**NOT (Title)** (cardiomyopathy OR “smooth muscle” OR systolic OR diastolic)

**NOT (Title)** (“in vitro” OR “in-vitro” OR C2C12)

**Query box:**

(((((((((peptide[Title/Abstract] OR "hormonal peptide"[Title/Abstract] OR polypeptide*[Title/Abstract] OR oligopeptide*[Title/Abstract] OR peptide*[Title/Abstract] OR "peptide chain*"[Title/Abstract] OR "hormonal peptide*"[Title/Abstract] OR "hormone peptide*"[Title/Abstract] OR "hormone, peptide*"[Title/Abstract] OR "peptide hormone*"[Title/Abstract] OR "peptide, hormonal*"[Title/Abstract] OR "polypeptide hormone*"[Title/Abstract] OR "chain, polypeptide*"[Title/Abstract] OR "peptide, poly"[Title/Abstract] OR "polypeptide chain*"[Title/Abstract] OR "peptide, oligo"[Title/Abstract] OR "bioactive peptide*"[Title/Abstract] OR "therapeutic peptide*"[Title/Abstract] OR "therapeutical peptide*"[Title/Abstract] OR "bacterial peptide*"[Title/Abstract] OR "exogenous peptide*"[Title/Abstract] OR "endogenous peptide*"[Title/Abstract] OR "natural peptide*"[Title/Abstract])) AND ((human[Text Word] OR trial[Text Word] OR RCT[Text Word] OR "first in human study"[Text Word] OR patient*[Text Word] OR child*[Text Word] OR animal*[Text Word] OR "animal experiment*"[Text Word] OR rodent[Text Word] OR "murine model"[Text Word] OR "rat"[Text Word] OR "rats"[Text Word] OR "mouse"[Text Word] OR "mouse model"[Text Word] OR "rat model"[Text Word] OR primate[Text Word] OR "Caenorhabditis elegans"[Text Word] OR Drosophila[Text Word] OR "zebra fish"[Text Word] OR human*[Text Word] OR "Homo sapiens"[Text Word] OR "human being*"[Text Word] OR "human body"[Text Word] OR "human race*"[Text Word] OR "human subject*"[Text Word] OR man[Text Word] OR "first-in-human study"[Text Word] OR "first-in-man study"[Text Word] OR sufferer*[Text Word] OR participant*[Text Word] OR men[Text Word] OR man[Text Word] OR woman[Text Word] OR women[Text Word] OR rodent*[Text Word] OR "murin model*"[Text Word] OR Rattus[Text Word] OR mice[Text Word] OR Mus[Text Word] OR "newborn mice"[Text Word] OR "animal population group*"[Text Word] OR Animalia[Text Word] OR Metazoa[Text Word] OR metazoan*[Text Word] OR metazoon[Text Word] OR "animal experimentation"[Text Word] OR "animal physical conditioning"[Text Word] OR "animal studies"[Text Word] OR "animal study"[Text Word] OR "animal trial*"[Text Word] OR "experiment, animal"[Text Word] OR "physical conditioning, animal"[Text Word] OR primate*[Text Word] OR "C elegans"[Text Word] OR "C. elegans"[Text Word] OR nematode[Text Word] OR "Rhabditis elegans"[Text Word] OR roundworm[Text Word] OR drosophile[Text Word] OR Drosophilia[Text Word] OR "fruit fly"[Text Word] OR "Brachydanio rerio"[Text Word] OR "Cyprinus rerio"[Text Word] OR "Danio frankei"[Text Word] OR "Danio rerio"[Text Word] OR "zebra danio"[Text Word] OR zebrafish[Text Word] OR "zebra fish"[Text Word]))) AND ((sarcopenia[Title/Abstract] OR "muscle function"[Title/Abstract] OR "muscle strength"[Title/Abstract] OR "muscle mass"[Title/Abstract] OR "grip strength"[Title/Abstract] OR "gait speed"[Title/Abstract] OR "walking speed"[Title/Abstract] OR "physical function*"[Title/Abstract] OR "physical functioning"[Title/Abstract] OR "physical performance*"[Title/Abstract] OR cachexia[Title/Abstract] OR "muscle atrophy"[Title/Abstract] OR sarcopenic[Title/Abstract] OR "muscle function*"[Title/Abstract] OR "function, muscle"[Title/Abstract] OR "muscle performance*"[Title/Abstract] OR "muscular effort*"[Title/Abstract] OR "muscular function*"[Title/Abstract] OR "muscular performance*"[Title/Abstract] OR "muscle work"[Title/Abstract] OR "muscular work"[Title/Abstract] OR "performance, muscle"[Title/Abstract] OR "work, muscle"[Title/Abstract] OR "dynamic strength, muscle"[Title/Abstract] OR "dynamic strength, muscular"[Title/Abstract] OR "force, muscle"[Title/Abstract] OR "muscle dynamic strength"[Title/Abstract] OR "muscle force"[Title/Abstract] OR "muscle force velocity relationship"[Title/Abstract] OR "muscle power"[Title/Abstract] OR "muscular dynamic strength"[Title/Abstract] OR "muscular force"[Title/Abstract] OR "muscular power"[Title/Abstract] OR "strength, muscle"[Title/Abstract] OR "muscular strength"[Title/Abstract] OR "muscle volume"[Title/Abstract] OR "muscle weight"[Title/Abstract] OR "weight, muscle"[Title/Abstract] OR "grasp force*"[Title/Abstract] OR "grasp strength"[Title/Abstract] OR "grasping strength"[Title/Abstract] OR "gripping force"[Title/Abstract] OR "gripping power"[Title/Abstract] OR "handgrip strength"[Title/Abstract] OR "ability, physical"[Title/Abstract] OR "assessment, physical performance*"[Title/Abstract] OR "evaluation, physical performance*"[Title/Abstract] OR "performance, physical"[Title/Abstract] OR "physical performance assessment*"[Title/Abstract] OR "physical performance evaluation*"[Title/Abstract] OR "physical performancy"[Title/Abstract] OR "cachectic state"[Title/Abstract] OR "cancer cachexia"[Title/Abstract] OR cachexia[Title/Abstract] OR cachectic[Title/Abstract] OR amyotrophia[Title/Abstract] OR amyotrophy[Title/Abstract] OR "atrophic muscular disorders"[Title/Abstract] OR "atrophy type 2"[Title/Abstract] OR "atrophy, muscle"[Title/Abstract] OR "degeneration, muscle"[Title/Abstract] OR "muscle atrophia"[Title/Abstract] OR "muscle cell degeneration"[Title/Abstract] OR "muscle degeneration"[Title/Abstract] OR "muscle fiber atrophy"[Title/Abstract] OR "muscle fiber degeneration"[Title/Abstract] OR "muscle recession"[Title/Abstract] OR "muscle wasting"[Title/Abstract] OR "muscular atrophy"[Title/Abstract] OR "muscular degeneration"[Title/Abstract] OR "muscular disorders, atrophic"[Title/Abstract] OR myoatrophy[Title/Abstract] OR myodegeneration[Title/Abstract] OR "myofibrillar degeneration"[Title/Abstract] OR myophagism[Title/Abstract]))) NOT ((dystrophia[Title] OR "dystrophic syndrome"[Title] OR dystrophy[Title] OR "congenital atonic sclerotic muscular dystrophy"[Title] OR "congenital muscle dystrophy"[Title] OR "congenital muscular dystrophy"[Title] OR "dystrophy, muscle"[Title] OR "muscle dystrophia"[Title] OR "muscle dystrophy"[Title] OR "muscular dystrophia"[Title] OR "muscular dystrophies"[Title] OR "muscular dystrophy, congenital"[Title] OR myodystrophia[Title] OR myodystrophy[Title] OR "muscular dystrophy"[Title] OR "neurogenic muscle disease"[Title] OR "neurogenic myopathy"[Title] OR "neuromuscular diseases"[Title] OR "neuromuscular disorder"[Title] OR "neuromuscular dysfunction"[Title] OR "neuromuscular syndrome"[Title] OR neuromyopathy[Title] OR "neuromuscular disease"[Title] OR myasthenia[Title] OR ALS[Title] OR "amyotrophic lateral sclerosis"[Title] OR "allergic myositis"[Title] OR "idiopathic inflammatory myopathy"[Title] OR "muscle infection"[Title] OR "myositis, allergic"[Title] OR neuromyositis[Title] OR myositis[Title] OR "Spinal muscular atrophy"[Title] OR SMA[Title]))) NOT ((cardiomyopathy[Title] OR "smooth muscle"[Title] OR systolic[Title] OR diastolic[Title]))) NOT (("in vitro"[Title] OR "in-vitro"[Title] OR C2C12[Title]))) AND ((Journal Article[Publication Type] OR Letter[Publication Type] OR Preprint[Publication Type]))) NOT ((Review[Publication Type] OR Systematic Review[Publication Type] OR Meta-analysis[Publication Type] OR Case Reports[Publication Type] OR Editorial[Publication Type]))) AND ((english[Language]))

(number of references: 1159)

1. **Web of Science** [Last searched August 6, 2023]

**(Topic)**

(peptide OR “hormonal peptide” OR polypeptide* OR oligopeptide* OR peptide* OR “peptide* chain*” OR “hormonal* peptide*” OR “hormone* peptide*” OR “hormone*, peptide*” OR “peptide* hormone*” OR “peptide*, hormonal*” OR “polypeptide* hormone*” OR “chain, polypeptide*” OR “peptide*, poly” OR “polypeptide* chain*” OR “peptide*, oligo” OR “bioactive peptide*” OR “therapeutic* peptide*” OR “bacterial* peptide*” OR “exogenous peptide*” OR “endogenous peptide*” OR “natural peptide*”)

**AND** **(Topic)**

(human OR trial OR RCT OR “first in human study” OR patient* OR child* OR animal* OR “animal experiment*” OR rodent OR “murine model” OR rat* OR mouse OR “mouse model” OR “rat model” OR primate OR “Caenorhabditis elegans” OR Drosophila OR “zebra fish” OR human* OR “Homo sapiens” OR “human being*” OR “human body” OR “human race*” OR “human subject*” OR man OR “first-in-human study” OR “first-in-man study” OR sufferer* OR participant* OR men OR man OR woman OR women OR rodent* OR “murin* model*” OR Rattus OR mice OR Mus OR “newborn mice” OR “animal population group*” OR Animalia OR Metazoa OR metazoan* OR metazoon OR “animal* experimentation” OR “animal* physical conditioning” OR “animal studies” OR “animal study” OR “animal trial*” OR “experiment*, animal” OR “physical conditioning, animal” OR primate* OR “C elegans” OR “C. elegans” OR nematode OR “Rhabditis elegans” OR roundworm OR drosophile OR Drosophilia OR “fruit fly” OR “Brachydanio rerio” OR “Cyprinus rerio” OR “Danio frankei” OR “Danio rerio” OR “zebra danio” OR zebrafish OR “zebra fish”)

**AND** **(Topic)**

(sarcopenia OR “muscle function” OR “muscle strength” OR “muscle mass” OR “grip strength” OR “gait speed” OR “walking speed” OR “physical function*” OR “physical functioning” OR “physical performance*” OR cachexia OR “muscle atrophy” OR sarcopenic OR “muscle function*” OR “function*, muscle” OR “muscle performance*” OR “muscular effort*” OR “muscular function*” OR “muscular performance*” OR “muscle work” OR “muscular work” OR “performance*, muscle” OR “work, muscle” OR “dynamic strength, muscle” OR “dynamic strength, muscular” OR “force, muscle” OR “muscle dynamic strength” OR “muscle force” OR “muscle force velocity relationship” OR “muscle power” OR “muscular dynamic strength” OR “muscular force” OR “muscular power” OR “strength, muscle” OR “muscular strength” OR “muscle volume” OR “muscle weight” OR “weight, muscle” OR “grasp force*” OR “grasp strength” OR “grasping strength” OR “gripping force” OR “gripping power” OR “handgrip strength” OR “abilit*, physical” OR “assessment*, physical performance*” OR “evaluation*, physical performance*” OR “performance*, physical” OR “physical performance* assessment*” OR “physical performance* evaluation*” OR “physical performancy” OR “cachectic state” OR “cancer cachexia” OR cachexia OR cachectic OR amyotrophia OR amyotrophy OR “atrophic muscular disorders” OR “atrophy type 2” OR “atrophy, muscle” OR “degeneration, muscle” OR “muscle atrophia” OR “muscle cell degeneration” OR “muscle degeneration” OR “muscle fiber atrophy” OR “muscle fiber degeneration” OR “muscle recession” OR “muscle wasting” OR “muscular atrophy” OR “muscular degeneration” OR “muscular disorders, atrophic” OR myoatrophy OR myodegeneration OR “myofibrillar degeneration” OR myophagism)

**NOT (Title)**

(dystrophia OR “dystrophic syndrome” OR dystrophy OR “congenital atonic sclerotic muscular dystrophy” OR “congenital muscle dystrophy” OR “congenital muscular dystrophy” OR “dystrophy, muscle” OR “muscle dystrophia” OR “muscle dystrophy” OR “muscular dystrophia” OR “muscular dystrophies” OR “muscular dystrophy, congenital” OR myodystrophia OR myodystrophy OR “muscular dystrophy” OR “neurogenic muscle disease” OR “neurogenic myopathy” OR “neuromuscular diseases” OR “neuromuscular disorder” OR “neuromuscular dysfunction” OR “neuromuscular syndrome” OR neuromyopathy OR “neuromuscular disease” OR myasthenia OR ALS OR “amyotrophic lateral sclerosis” OR “allergic myositis” OR “idiopathic inflammatory myopathy” OR “muscle infection” OR “myositis, allergic” OR neuromyositis OR myositis OR “Spinal muscular atrophy” OR SMA)

**NOT (Title)**

(cardiomyopathy OR “smooth muscle” OR systolic OR diastolic)

**NOT (Title)**

(“in vitro” OR “in-vitro” OR C2C12)

**AND (Language of article)**

“english”

**AND (Publication type)**

(“Article” OR “Letter”)

**NOT (Publication type)**

(“review” OR “retracted publication” OR “book chapter” OR ''meeting abstract” OR “editorial”)

(number of references: 1583)

In total 3857 references

After duplicate removal: 1815 references
