# Supplementary material for: Role of Peptides in Skeletal Muscle Wasting: A Scoping Review
Source: J Cachexia Sarcopenia Muscle. 2025 Nov 13;16(6):e70109. doi: 10.1002/jcsm.70109 (PMC12613835; doi:10.1002/jcsm.70109)
Supplement: Supplementary file 3 — Data S3: Component‐level summary of muscle outcomes (muscle mass, strength, physical performance or composite sarcopenia) associated with each peptide. [file JCSM-16-e70109-s004.docx]

Supporting File S3. Component-level summary of muscle outcomes (muscle mass, strength, physical performance or composite sarcopenia) associated with each peptide. Values represent the number of studies reporting an association.

| **Peptide name** | **Effect on muscle wasting endpoint** | | | | | | | | | | | |
| --- | --- | --- | --- | --- | --- | --- | --- | --- | --- | --- | --- | --- |
|  | **Muscle mass** | | | **Muscle strength** | | | **Physical performance** | | | **Sarcopenia** | | |
|  | **Beneficial** | **Detrimental** | **Neutral** | **Beneficial** | **Detrimental** | **Neutral** | **Beneficial** | **Detrimental** | **Neutral** | **Beneficial** | **Detrimental** | **Neutral** |
| Gly-Pro-Hyp | **1** | 0 | 0 | 0 | 0 | **1** | 0 | 0 | 0 | 0 | 0 | 0 |
| SS-31 | 3 | 0 | 2 | 6 | 0 | 1 | 4 | 0 | 1 | 0 | 0 | 0 |
| Cblin | 2 | 0 | 0 | 0 | 0 | 0 | 0 | 0 | 0 | 0 | 0 | 0 |
| C14 Cblin | 1 | 0 | 0 | 0 | 0 | 0 | 0 | 0 | 0 | 0 | 0 | 0 |
| BIM-28131 | 3 | 0 | 0 | 0 | 0 | 0 | 0 | 0 | 0 | 0 | 0 | 0 |
| TCMCB07 | 1 | 0 | 1 | 0 | 0 | 0 | 0 | 0 | 0 | 0 | 0 | 0 |
| Substituted ACTH (4-9)# | 0 | 0 | 0 | 1 | 0 | 0 | 0 | 0 | 0 | 0 | 0 | 0 |
| C6M | 0 | 1 | 0 | 0 | 0 | 1 | 0 | 0 | 0 | 0 | 0 | 0 |
| HNK-1 mimetic peptide | 0 | 0 | 0 | 1 | 0 | 0 | 1 | 0 | 0 | 0 | 0 | 0 |
| [pyr1] Apelin 13 | 0 | 0 | 0 | 1 | 0 | 0 | 1 | 0 | 0 | 0 | 0 | 0 |
| D-Apelin 13 | 0 | 0 | 1 | 0 | 0 | 0 | 0 | 0 | 0 | 0 | 0 | 0 |
| IC6 | 1 | 0 | 0 | 0 | 0 | 1 | 0 | 0 | 0 | 0 | 0 | 0 |
| Cblin-like-peptide | 1 | 0 | 0 | 0 | 0 | 0 | 0 | 0 | 0 | 0 | 0 | 0 |
| Melanocyte-stimulating hormone alpha (a-MSH) | 0 | 0 | 0 | 0 | 1 | 0 | 0 | 0 | 0 | 0 | 0 | 0 |
| Proctolin | 0 | 0 | 0 | 1 | 0 | 0 | 0 | 0 | 0 | 0 | 0 | 0 |
| Angiotensin (1-7) | 1 | 0 | 1 | 5 | 0 | 0 | 1 | 0 | 1 | 0 | 0 | 0 |
| Substance-P | 0 | 0 | 0 | 0 | 1 | 0 | 0 | 0 | 0 | 0 | 0 | 0 |
| ARA 284 | 1 | 0 | 0 | 0 | 0 | 0 | 0 | 0 | 0 | 0 | 0 | 0 |
| FMRF-a | 0 | 0 | 0 | 1 | 0 | 0 | 0 | 0 | 0 | 0 | 0 | 0 |
| Angiotensin II | 0 | 1 | 0 | 0 | 1 | 1 | 0 | 0 | 1 | 0 | 0 | 0 |
| Acein | 0 | 0 | 0 | 0 | 0 | 0 | 1 | 0 | 0 | 0 | 0 | 0 |
| YPLP | 0 | 0 | 0 | 0 | 0 | 0 | 1 | 0 | 0 | 0 | 0 | 0 |
| DF-3 | 1 | 0 | 0 | 0 | 0 | 0 | 0 | 0 | 0 | 0 | 0 | 0 |
| ACTH (4- 10) | 0 | 0 | 0 | 1 | 0 | 0 | 0 | 0 | 0 | 0 | 0 | 0 |
| Growth hormone releasing peptide 2 (GHRP-2) | 0 | 0 | 1 | 0 | 0 | 0 | 0 | 0 | 0 | 0 | 0 | 0 |
| Rubiscolin-6 | 0 | 0 | 0 | 0 | 0 | 0 | 1 | 0 | 0 | 0 | 0 | 0 |
| iAM373 | 0 | 1 | 0 | 0 | 0 | 1 | 0 | 2 | 0 | 0 | 0 | 0 |
| Acylated Ghrelin | 7 | 1 | 4 | 4 | 0 | 2 | 1 | 0 | 2 | 0 | 1 | 2 |
| MZ-5-156 | 0 | 0 | 0 | 0 | 0 | 1 | 1 | 0 | 0 | 0 | 0 | 0 |
| ACTH 1-17 | 0 | 0 | 0 | 1 | 0 | 0 | 0 | 0 | 0 | 0 | 0 | 0 |
| Unacylated Ghrelin | 0 | 0 | 0 | 0 | 0 | 0 | 0 | 0 | 0 | 0 | 2 | 0 |
| MOTS-c | 6 | 0 | 0 | 4 | 0 | 0 | 3 | 0 | 0 | 0 | 0 | 0 |
| MID-35 | 3 | 0 | 0 | 1 | 0 | 0 | 0 | 0 | 0 | 0 | 0 | 0 |
| MIPE-1686 | 1 | 0 | 0 | 0 | 0 | 0 | 0 | 0 | 0 | 0 | 0 | 0 |
| Peptide-2 (from mouse myostatin precursor) | 1 | 0 | 0 | 1 | 0 | 0 | 0 | 0 | 0 | 0 | 0 | 0 |
| CSP-7 | 0 | 1 | 0 | 0 | 1 | 0 | 0 | 0 | 0 | 0 | 0 | 0 |
| [K15,R16,L27]VIP(1-7)/GRF(8-27) | 0 | 0 | 2 | 0 | 0 | 0 | 0 | 0 | 0 | 0 | 0 | 0 |
| GLP-1 (7-36) | 1 | 1 | 0 | 1 | 0 | 0 | 0 | 1 | 0 | 0 | 3 | 0 |
| Obestatin | 0 | 0 | 0 | 0 | 1 | 0 | 0 | 1 | 0 | 0 | 0 | 0 |
| MBP-Pro45-70-His6 | 1 | 0 | 0 | 1 | 0 | 0 | 1 | 0 | 0 | 0 | 0 | 0 |
| Pep45-65-NH2 | 1 | 0 | 0 | 1 | 0 | 0 | 1 | 0 | 0 | 0 | 0 | 0 |
| N Terminal peptide of the PIF receptor (D-version) | 1 | 0 | 0 | 0 | 0 | 0 | 0 | 0 | 0 | 0 | 0 | 0 |
| Humanin | 0 | 0 | 0 | 0 | 1 | 0 | 0 | 0 | 0 | 0 | 0 | 0 |
| Endothelin-1 | 0 | 1 | 0 | 0 | 1 | 0 | 0 | 1 | 0 | 0 | 0 | 0 |
| NF-kappaB inhibitor peptide (NBD peptide) | 1 | 0 | 0 | 0 | 0 | 0 | 0 | 0 | 0 | 0 | 0 | 0 |
| Apelin | 3 | 0 | 1 | 2 | 1 | 1 | 4 | 0 | 1 | 0 | 0 | 0 |
| Peptide YY | 0 | 0 | 0 | 0 | 1 | 0 | 0 | 0 | 0 | 0 | 1 | 1 |
| Neuropeptide Y | 1 | 0 | 0 | 0 | 0 | 0 | 0 | 0 | 0 | 0 | 0 | 0 |
| Crotamine | 0 | 0 | 1 | 0 | 0 | 0 | 0 | 0 | 0 | 0 | 0 | 0 |
| Adropin | 0 | 1 | 0 | 0 | 0 | 0 | 0 | 0 | 0 | 0 | 0 | 0 |
| Ro-25-1553 (cyclic) | 3 | 0 | 0 | 1 | 0 | 0 | 0 | 0 | 0 | 0 | 0 | 0 |
| [P11L12L13E22K23A35R36A39R40] Urocortin I | 1 | 0 | 0 | 0 | 0 | 0 | 0 | 0 | 0 | 0 | 0 | 0 |
| [P11L12L13E22K23A35Q36A39Q40] Urocortin I | 1 | 0 | 0 | 0 | 0 | 0 | 0 | 0 | 0 | 0 | 0 | 0 |
| Glucose-dependent insulinotropic polypeptide (GIP) | 0 | 1 | 0 | 0 | 0 | 0 | 0 | 0 | 0 | 0 | 0 | 0 |
| GHRH-1,44-amide | 0 | 0 | 0 | 0 | 0 | 1 | 1 | 0 | 0 | 0 | 0 | 0 |
| [V10P11L12F13E22K23A35R36A39R40] Urocortin I | 1 | 0 | 0 | 0 | 0 | 0 | 0 | 0 | 0 | 0 | 0 | 0 |
| [P11T12Y13]Sauvagine | 1 | 0 | 0 | 0 | 0 | 0 | 0 | 0 | 0 | 0 | 0 | 0 |
| [P11T12W13]Sauvagine | 1 | 0 | 0 | 0 | 0 | 0 | 0 | 0 | 0 | 0 | 0 | 0 |
| [V10P11L12L13E22K23A35Q36A39Q40] Urocortin I | 1 | 0 | 0 | 0 | 0 | 0 | 0 | 0 | 0 | 0 | 0 | 0 |
| Exendin-4 | 3 | 0 | 0 | 3 | 0 | 0 | 0 | 0 | 0 | 0 | 0 | 0 |
| [P11F12Q13]Sauvagine | 1 | 0 | 0 | 0 | 0 | 0 | 0 | 0 | 0 | 0 | 0 | 0 |
| [P11L12B13]Sauvagine | 1 | 0 | 0 | 0 | 0 | 0 | 0 | 0 | 0 | 0 | 0 | 0 |
| Midregional pro-Adrenomedullin (MR-proADM) | 0 | 1 | 0 | 0 | 0 | 0 | 0 | 0 | 0 | 0 | 0 | 0 |
| [P11I12Q13]Sauvagine | 1 | 0 | 0 | 0 | 0 | 0 | 0 | 0 | 0 | 0 | 0 | 0 |
| [P11Q12I13]Sauvagine | 1 | 0 | 0 | 0 | 0 | 0 | 0 | 0 | 0 | 0 | 0 | 0 |
| Osteocalcin | 0 | 1 | 2 | 0 | 0 | 0 | 0 | 0 | 0 | 0 | 0 | 0 |
| [P11I12Y13]Sauvagine | 1 | 0 | 0 | 0 | 0 | 0 | 0 | 0 | 0 | 0 | 0 | 0 |
| Alpha-defensin 26 | 0 | 1 | 0 | 0 | 0 | 0 | 0 | 0 | 0 | 0 | 0 | 0 |
| Sauvagine | 2 | 0 | 0 | 0 | 0 | 0 | 0 | 0 | 0 | 0 | 0 | 0 |
| Brain natriuretic peptide | 0 | 4 | 0 | 0 | 5 | 2 | 0 | 4 | 0 | 0 | 3 | 0 |
| [P11I12L13]Sauvagine | 1 | 0 | 0 | 0 | 0 | 0 | 0 | 0 | 0 | 0 | 0 | 0 |
| Urocortin I | 2 | 0 | 0 | 0 | 0 | 0 | 0 | 0 | 0 | 0 | 0 | 0 |
| Midregional pro-Atrial natriuretic peptide (MR-proANP) | 0 | 1 | 0 | 0 | 0 | 0 | 0 | 0 | 0 | 0 | 0 | 0 |
| GLP-2 | 1 | 0 | 1 | 1 | 0 | 0 | 0 | 0 | 0 | 0 | 0 | 0 |
| Copeptin | 0 | 0 | 1 | 0 | 0 | 0 | 0 | 0 | 0 | 0 | 0 | 0 |
| Alpha-defensin 5 | 0 | 0 | 1 | 0 | 0 | 0 | 0 | 0 | 0 | 0 | 0 | 0 |
| Compound B ([E22K23Q24E25K26E27K28Q29]hUCN2) | 2 | 0 | 0 | 1 | 0 | 0 | 0 | 0 | 0 | 0 | 0 | 0 |
| C-peptide | 11 | 1 | 0 | 1 | 0 | 1 | 0 | 0 | 2 | 2 | 0 | 0 |
| ACTH (1-39) | 0 | 0 | 0 | 1 | 0 | 0 | 0 | 0 | 0 | 0 | 0 | 0 |
| Compound A ([E22K23Q24E25K26E27K28C29]hUCN2) | 3 | 0 | 0 | 1 | 0 | 1 | 0 | 0 | 0 | 0 | 0 | 0 |
| Alpha-calcitonin gene-related peptide (a-CGRP) | 0 | 0 | 0 | 1 | 0 | 0 | 0 | 0 | 0 | 0 | 0 | 0 |
| Insulin | 9 | 0 | 1 | 0 | 2 | 1 | 1 | 0 | 2 | 0 | 1 | 0 |
| Abaloparatide (ABL) | 0 | 0 | 1 | 0 | 0 | 0 | 0 | 0 | 0 | 0 | 0 | 0 |
| Urocortin II | 4 | 0 | 0 | 2 | 0 | 0 | 0 | 0 | 0 | 0 | 0 | 0 |
| MS 9a-1 | 0 | 0 | 0 | 1 | 0 | 0 | 0 | 0 | 0 | 0 | 0 | 0 |
| Bim-28125 | 1 | 0 | 0 | 0 | 0 | 0 | 0 | 0 | 0 | 0 | 0 | 0 |
| Type I collagen carboxyl-terminal peptide β glypeptide (β-CTX) | 0 | 1 | 0 | 0 | 0 | 0 | 0 | 0 | 0 | 0 | 0 | 0 |
| **Total** | **96** | **19** | **21** | **46** | **16** | **16** | **23** | **9** | **10** | **2** | **11** | **3** |
