# Supplementary material for: Role of Peptides in Skeletal Muscle Wasting: A Scoping Review
Source: J Cachexia Sarcopenia Muscle. 2025 Nov 13;16(6):e70109. doi: 10.1002/jcsm.70109 (PMC12613835; doi:10.1002/jcsm.70109)
Supplement: Supplementary file 4 — Data S4: Presumed muscle wasting targets and downstream processes of the peptides. [file JCSM-16-e70109-s002.docx]

**Supporting File S4: Presumed muscle wasting targets and downstream processes of the identified peptides**^1–62^

**Urocortin I** and **II** are neuropeptides belonging to the corticotropin-releasing hormone (CRH) family, sharing an evolutionary lineage with the non-mammalian peptide **sauvagine**. These neuropeptides, along with their derivatives, bind to the G-protein coupled receptor corticotropin-releasing factor type 2 receptor (CRF2R), initiating anabolic and anti-catabolic effects in skeletal muscle. Their mechanisms likely involve activation of the cyclic adenosine monophosphate (cAMP) cascade and downstream participation of key signaling pathways, including protein kinase A (PKA), Akt, and ERK1/2^S1^. Other neuropeptides with direct effects include neuropeptide hormones **Angiotensin II** and **Angiotensin (1-7)**, which operate antagonistically. Ang II binds to the AT1 receptor (AT1R), which inhibits the PI3K/Akt/mTOR pathway, while Ang (1-7) binds to the Mas receptor (MasR), promoting the same pathway^S2^. Furthermore, angiotensin II also enhances nicotinamide adenine dinucleotide phosphate (NADPH) oxidase activity leading to increased production of reactive oxygen species (ROS)^S3^.  **Apelin** is an exerkine, i.e. an exercise-induced signaling molecule, with the Apelin/APJ (apelin receptor)/AMPK axis as one of the major underlying mechanisms regulating the expression of muscle-based thermogenic genes and stimulating mitochondrial biogenesis^S4^. Apelin also activates the PI3K/AKT pathway in myofibers and stimulates muscle stem cells, thereby reducing the damage caused by oxidative stress and inflammation^S5-S6^. Growth hormone-releasing hormone (GHRH) and its derivatives, such as **GHRH1-44 amide**, elevate serum insulin-like growth factor 1 (IGF-1) levels, which in turn stimulate the PI3K/Akt/mTOR pathway^S7^. The non-mammalian neuropeptides **Proctolin** and FMRFamide (**FMRFa**) stimulate muscle contractions by promoting calcium release from the sarcoplasmic reticulum as well as activating calcium-dependent proteins such as Ca2+/calmodulin-dependent protein kinases^S8-S9^. Adrenocorticotropic hormone (ACTH) fragments **ACTH 4-9, 4-10,** and **1-39** increase the formation, maintenance, and organization of motor units during both muscle development and reinnervation, although the exact mechanism is unclear^S10^.

Neuropeptides with indirect muscle effects include **Substance P**, a multifaceted compound which increases fibroblast migration and collagen synthesis in the extracellular muscle region, leading to fibrosis and decreased muscle quality^S11^. The spinach-derived opioid peptide **Rubiscolin-6** has been shown to improve locomotion in mice with cancer cachexia. Although its precise mechanism remains unclear, rubiscolin-6 likely influences dopaminergic neurons and neuropeptide Y (NPY), thereby enhancing both mobility and appetite-stimulating effects. Additionally, rubiscolin-6 increases the expression of AMPK and glucose transporter 4 (GLUT4) in muscle cells, which may support improved muscle function. Its analgesic properties are also thought to contribute positively to locomotion^S12-S13^. **Acein** is a *de-novo* synthesized peptide which improves physical performance in *C. elegans* by inhibiting C-type lectin domain-containing protein (CLEC-126) and stimulating dopamine secretion. **Mz-5-156**, a GHRH antagonist, enhances physical performance by improving central muscle coordination^S14^. **TCMCB07** is a synthetic antagonist of the melanocortin 4 receptor (MC4R), stimulating appetite and having an orexigenic effect^S15^. Similarly, the snake venom **crotamine** induces skeletal muscle spasms mediated by interactions with sodium and potassium channels^S16^. However, no effect on muscle wasting has been observed^S17^. **MS 9a-1** is a positive allosteric modulator of transient receptor potential ankyrin 1 (TRPA1), an ion channel in sensory neurons involved in inflammation and pain. By enhancing TRPA1 activation, MS 9a-1 induces desensitization, reducing musculoskeletal pain and inflammation, ultimately improving strength^S18^. Alpha calcitonin gene-related peptide (**αCGRP**) affects skeletal muscle function through the neuromuscular junction, where it is co-expressed with acetylcholine (Ach) in motor neurons, stimulating Ach receptor synthesis, and decreasing acetylcholinesterase levels^S19^. **Endothelin-1** (ET-1) is a potent vasoconstrictor peptide reducing insulin-stimulated myoblast differentiation through Endothelin type B (ETB) receptors and the p38 MAPK dependent pathway^S20^. **BNP**, mid-regional pro-atrial natriuretic peptide (**MR-proANP**), mid-regional pro-adrenomedullin (**MR-proADM**) and **copeptin**, explored as peptide biomarkers for heart disease, are associated with decreased muscle mass (BNP, MR-proANP and MR-proADM) or are muscle neutral (copeptin). They are not connected to a specific biochemical pathway as there is no current evidence of their direct involvement in muscle homeostasis^S21^.

Gastrointestinal (GI) and pancreatic peptides also commonly demonstrate bioactivity towards skeletal muscles. Glucagon-like peptide 1 (**GLP-1**) and its derivatives bind to the GLP-1 receptor (GLP-1R) on muscle cells, activating adenylate cyclase and increasing cAMP levels, followed by activation of PKA. PKA subsequently activates the PI3K/Akt/mTOR pathway, leading to decreased expression of the ubiquitin ligases MuRF-1 and atrogin-1, thereby reducing muscle atrophy^S22^. **Exendin-4**, a GLP-1R agonist, increases mRNA expression of thermogenic genes via cAMP/PKA signaling in skeletal muscle cells^S23^. Glucagon-like peptide 2 (**GLP-2**) attenuates the decline of skeletal muscle strength and weight via activation of the GLP-2 receptor (GLP-2R)-PI3K/Akt/FOXO3a phosphorylation pathway^S24^. Insulin primarily acts through the insulin receptor (IR)-PI3K/Akt pathway^S25^. **C-peptide** muscle signaling is believed to involve yet unidentified receptors that stimulate the ERK1/2 and AMPK pathways^S26-S28^. **Obestatin** promotes myogenic differentiation and fusion of human myoblasts through G protein-coupled receptor 39 (GPR39)^S29^. **Ro-25-1553** is a derivative of vasoactive intestinal peptide (**VIP**) that selectively acts on vasoactive intestinal peptide receptor 2 (VPAC2R) expressed on skeletal muscle, activating the MAPK pathway^S30^. The muscle-beneficial effects of Ro-25-1553 are also believed to occur through increased muscular IGF-1 levels and facilitation of Ach release from motor nerve terminals. **[K^15^,R^16^,L^27^]VIP(1-7)/GRF(8-27)** is a vasoactive intestinal polypeptide receptor 1 (VPAC1R) selective agonist which shows no beneficial clinical effects on muscle wasting, demonstrating the skeletal muscle-specific effects of VPAC2R activation^S30^. **Ghrelin** and its derivatives **GHRP-2**, **Bim-28125** and **Bim-28131** activate the MAPK-ERK1/2 signaling as well as Akt signaling through the growth hormone secretagogue receptor 1a (GHS-R1a) as well as having GHSR-independent effects on muscle cells^S31-S33^. Additionally, ghrelin indirectly influences muscle function through reduction of inflammatory cytokines by inhibiting the nucleotide-binding domain, leucine-rich repeat, and pyrin domain-containing protein 3 (NLRP3) inflammasome^S34^. Other GI peptides have indirect muscle effects. **Peptide YY** (**PYY**) and **alpha-melanocyte-stimulating hormone (α-MSH)** (acting through MC4R) are associated with low muscle mass and strength in older populations through their central appetite-suppressing effects which lead to decreased available amino acids for muscle tropism^S35-S36^. **Glucose-dependent insulinotropic polypeptide** (**GIP**) receptor signaling promotes the differentiation of fibro-adipogenic progenitors into mature adipocytes in skeletal muscles and the formation of intramuscular adipose tissue thereby accelerating sarcopenia^S37^. **Neuropeptide Y** induces an orexigenic effect, and is positively correlated with muscle strength^S38^.

Myostatin is an endogenous negative regulator of skeletal muscle through binding to the activin receptor type 2B (ACTR2B) on skeletal muscle cells, which then recruits the activin receptor type 1B (ACTR1B). This receptor complex activates the SMAD2 and SMAD3 transcription factors, initiating signaling pathways that lead to muscle fiber atrophy^S39^. Follistatin is an endogenous antagonist of myostatin. Myostatin and follistatin derivatives **DF3**, **Peptide-2**, **MBP-Pro45-70-His6, Pep45-65, MID35** and **MIPE1686** have been designed to target these pathways and prevent muscle atrophy^S40-S42^.

Peptides influencing general cellular homeostasis pathways were also identified. Synthetic Casitas B-lineage lymphoma-b (Cbl-b) inhibitory peptides like **Cblin**, **C14 Cblin** and **Cblin-like peptide** inhibit the protein degradation effects of the ubiquitin E3 ligase Cbl-b^S43^. **Adropin** is a natural peptide present in multiple tissues increasing insulin induced Akt phosphorylation and GLUT4 expression^S44^. The NF‐κB essential modulator **(NEMO) binding domain (NBD) peptide** selectively blocks the activation of the IκB kinase (IKK) complex, inhibiting NF‐κB activation^S45^.

The extracellular matrix (ECM) of skeletal muscle contributes to muscle wasting through its role as a reservoir for growth factors like IGF-1, regulating their bioavailability by controlled trapping and release^S46^. Structurally, ECM proteins such as collagen provide elasticity and facilitate the transmission of contractile force. Beyond structural support, some collagen-derived peptides, such as **Gly-Pro-Hyp (GPH)**, exhibit bioactive properties, enhancing IGF-1 expression while downregulating myostatin, atrogin-1, MuRF, and FOXO3a in murine skeletal muscle^S47^. Other collagen peptides, including beta-C-terminal telopeptide of type I collagen (**β-CTX**), **IC6**, and **C6M**, are being investigated as biomarkers for muscle mass and anabolic/catabolic responses to muscle loading^S48^.

Mitochondria, which play a pivotal role in adenosine triphosphate (ATP) production and cellular energy balance, are emerging as important contributors to skeletal muscle homeostasis, leading to the exploration of mitochondrially derived and targeted peptides. Mitochondrially-derived peptides such as Mitochondrial ORF within Twelve S rRNA type-c (MOTS-c), humanin, short humanin like peptides 1 to 6 (SHLP1-6) and small human mitochondrial open reading frame over serine tRNA (SHMOOSE) have been linked to age-related cellular metabolism^S49^. **MOTS-c** enhances glucose uptake in skeletal muscle and improves insulin signaling. It activates both the AMPK/PGC-1α and Akt/mTOR pathways, with the latter being stimulated through the inhibition of Phosphatase and Tensin Homolog (PTEN) via Casein Kinase 2 (CK2). Additionally, MOTS-c directly suppresses the FOXO transcription factor, a key regulator of muscle atrophy^S50-S51^. The micropeptide **humanin** activates ERK1/2 signaling^S52^ and regenerative autophagy, responsible for eliminating damaged organelles and misfolded proteins^S53^. **SS-31** (elamipretide) is a synthetic peptide that selectively targets mitochondria by binding to cardiolipin, a phospholipid located in the inner mitochondrial membrane. The SS-31-cardiolipin complex inhibits cytochrome-C peroxidase activity, thereby preventing cardiolipin peroxidation and reducing the production of ROS^S54^.

Bacterial and fungal peptides, including those originating from the microbiome, also influence muscle homeostasis. The yeast derived peptide **YPLP** upregulates and activates AMPK, leading to increased expression of PGC-1α and improved mitochondrial function^S55^. The bacterial quorum sensing peptide **iAM373** decreases metabolic activity in muscle cells and impairs muscle function in *C. elegans* probably by disrupting pathways for muscle development and differentiation while increasing proteasomal degradation^S56^. Similarly, in mice, Competence Stimulating Peptide 7 (**CSP-7**) administration causes a decline in muscle mass and strength by stimulating chronic IL-6 release from muscle cells^S57^.

Immune system peptides include the antimicrobial peptides (AMPs) **α-defensin 5** (DEFA5) and **α-defensin** **26** (DEFA26). DEFA26 26 dietary supplementation influences intestinal microbiota, induces hypoinsulinemia, glucose intolerance and loss of lean muscle mass, while DEFA5 shows no effect on mice^S58^.

The remaining peptides could not be included in any of the previous groups. **ARA284** is an erythropoietin derivative activating the innate repair receptor (IRR) which reduces myostatin expression, induces Akt phosphorylation and GSK‐3β inhibition, leading to increased protein synthesis^S59^. **Osteocalcin**, primarily known for its role in skeletal homeostasis, promotes protein synthesis in mouse myotubes by direct activation of the Akt/mTOR pathway through GPC class C group 6A receptor (GPRC6A)^S60^. Other non-groupable peptides include **HNK-1-mimicking peptide,** believed to indirectly activate the receptor for advanced glycation end-products (RAGE) signaling pathway in neuronal cells^S61^ and **Abaloparatide** (ABL), a PTH related protein (PTHrP) analog showing no effect on skeletal muscle mass in mice^S62^.

**Supplementary References**^1–62^

S1. Lautherbach N, Gonçalves DAP, Silveira WA, et al. Urocortin 2 promotes hypertrophy and enhances skeletal muscle function through cAMP and insulin/IGF-1 signaling pathways. *Mol Metab*. 2022;60:101492.

S2. Rabie MA, Abd El Fattah MA, Nassar NN, El-Abhar HS, Abdallah DM. Angiotensin 1-7 ameliorates 6-hydroxydopamine lesions in hemiparkinsonian rats through activation of MAS receptor/PI3K/Akt/BDNF pathway and inhibition of angiotensin II type-1 receptor/NF-κB axis. *Biochem Pharmacol*. 2018;151:126-134.

S3. Wei Y, Sowers JR, Nistala R, et al. Angiotensin II-induced NADPH oxidase activation impairs insulin signaling in skeletal muscle cells. *J Biol Chem*. 2006;281(46):35137-35146.

S4. Son JS, Chae SA, Zhao L, et al. Maternal exercise intergenerationally drives muscle-based thermogenesis via activation of apelin-AMPK signaling. *EBioMedicine*. 2022;76:103842.

S5. Zheng XD, Huang Y, Li H. Regulatory role of Apelin-13-mediated PI3K/AKT signaling pathway in the glucose and lipid metabolism of mouse with gestational diabetes mellitus. *Immunobiology*. 2021;226(5):152135.

S6. Vinel C, Lukjanenko L, Batut A, et al. The exerkine apelin reverses age-associated sarcopenia. *Nat Med*. 2018;24(9):1360-1371.

S7. Sackmann-Sala L, Ding J, Frohman LA, Kopchick JJ. Activation of the GH/IGF-1 axis by CJC-1295, a long-acting GHRH analog, results in serum protein profile changes in normal adult subjects. *Growth Hormone & IGF Research*. 2009;19(6):471-477.

S8. Ormerod KG, Scibelli AE, Littleton JT. Regulation of excitation‐contraction coupling at the *Drosophila* neuromuscular junction. *J Physiol*. 2022;600(2):349-372.

S9. Ormerod KG, LePine OK, Bhutta MS, Jung J, Tattersall GJ, Mercier AJ. Characterizing the physiological and behavioral roles of proctolin in *Drosophila melanogaster*. *J Neurophysiol*. 2016;115(1):568-580.

S10. Saint-Come C, Acker GR, Strand FL. Development and regeneration of motor systems under the influence of ACTH peptides. *Psychoneuroendocrinology*. 1985;10(4):445-459.

S11. Fisher PW, Zhao Y, Rico MC, et al. Increased CCN2, substance P and tissue fibrosis are associated with sensorimotor declines in a rat model of repetitive overuse injury. *J Cell Commun Signal*. 2015;9(1):37-54.

S12. Kairupan TS, Cheng KC, Asakawa A, et al. Rubiscolin-6 activates opioid receptors to enhance glucose uptake in skeletal muscle. *J Food Drug Anal*. 2019;27(1):266-274.

S13. Iizasa E, Iwai H, Oyamada Y, et al. A plant-derived δ opioid receptor agonist rubiscolin-6 ameliorates sickness behavior in mice with cancer cachexia. *J Funct Foods*. 2024;119:106297.

S14. Banks WA, Morley JE, Farr SA, et al. Effects of a growth hormone-releasing hormone antagonist on telomerase activity, oxidative stress, longevity, and aging in mice. *Proceedings of the National Academy of Sciences*. 2010;107(51):22272-22277.

S15. Zhu X, Callahan MF, Gruber KA, Szumowski M, Marks DL. Melanocortin-4 receptor antagonist TCMCB07 ameliorates cancer- and chronic kidney disease–associated cachexia. *Journal of Clinical Investigation*. 2020;130(9):4921-4934.

S16. Oguiura N, Boni-Mitake M, Rádis-Baptista G. New view on crotamine, a small basic polypeptide myotoxin from South American rattlesnake venom. *Toxicon*. 2005;46(4):363-370.

S17. Marinovic MP, Campeiro JD, Lima SC, et al. Crotamine induces browning of adipose tissue and increases energy expenditure in mice. *Sci Rep*. 2018;8(1):5057.

S18. Maleeva EE, Palikova YA, Palikov VA, et al. Potentiating TRPA1 by Sea Anemone Peptide Ms 9a-1 Reduces Pain and Inflammation in a Model of Osteoarthritis. *Mar Drugs*. 2023;21(12):617.

S19. New H V., Mudge AW. Calcitonin gene-related peptide regulates muscle acetylcholine receptor synthesis. *Nature*. 1986;323(6091):809-811.

S20. Liu SY, Chen LK, Jhong YT, et al. Endothelin-1 impairs skeletal muscle myogenesis and development via ETB receptors and p38 MAPK signaling pathway. *Clin Sci*. 2024;138(12):711-723.

S21. Christensen HM, Kistorp C, Schou M, et al. Cross-talk between the heart and adipose tissue in cachectic heart failure patients with respect to alterations in body composition: A prospective study. *Metabolism*. 2014;63(1):141-149.

S22. Smith NK, Hackett TA, Galli A, Flynn CR. GLP-1: Molecular mechanisms and outcomes of a complex signaling system. *Neurochem Int*. 2019;128:94-105.

S23. Choung JS, Lee YS, Jun HS. Exendin-4 increases oxygen consumption and thermogenic gene expression in muscle cells. *J Mol Endocrinol*. 2017;58(2):79-90.

S24. Ye YL, Kuai Z, Qian DD, et al. GLP-2 ameliorates D-galactose induced muscle aging by IGF-1/Pi3k/Akt/FoxO3a signaling pathway in C2C12 cells and mice. *Arch Gerontol Geriatr*. 2024;124:105462.

S25. Glass DJ. PI3 Kinase Regulation of Skeletal Muscle Hypertrophy and Atrophy. In: ; 2010:267-278.

S26. Russo C, Lazzaro V, Gazzaruso C, et al. Proinsulin C-peptide modulates the expression of ERK1/2, type I collagen and RANKL in human osteoblast-like cells (Saos-2). *Mol Cell Endocrinol*. 2017;442:134-141.

S27. Maurotti S, Pujia R, Galluccio A, et al. Preventing muscle wasting: pro‐insulin C‐peptide prevents loss in muscle mass in streptozotocin‐diabetic rats. *J Cachexia Sarcopenia Muscle*. 2023;14(2):1117-1129.

S28. Bhatt MP, Lim YC, Kim YM, Ha KS. C-Peptide Activates AMPKα and Prevents ROS-Mediated Mitochondrial Fission and Endothelial Apoptosis in Diabetes. *Diabetes*. 2013;62(11):3851-3862.

S29. Santos-Zas I, Cid-Díaz T, González-Sánchez J, et al. Obestatin controls skeletal muscle fiber-type determination. *Sci Rep*. 2017;7(1):2137.

S30. Hinkle RT, Donnelly E, Cody DB, Sheldon RJ, Isfort RJ. Activation of the vasoactive intestinal peptide 2 receptor modulates normal and atrophying skeletal muscle mass and force. *J Appl Physiol*. 2005;98(2):655-662.

S31. Luo Q, Zhou Y, Chen MY, et al. Fasting up‐regulates ferroportin 1 expression via a Ghrelin/GHSR/MAPK signaling pathway. *J Cell Physiol*. 2018;233(1):30-37.

S32. Liu H, Zang P, Lee I, et al. Growth hormone secretagogue receptor‐1a mediates ghrelin’s effects on attenuating tumour‐induced loss of muscle strength but not muscle mass. *J Cachexia Sarcopenia Muscle*. 2021;12(5):1280.

S33. Tschöp M, Statnick MA, Suter TM, Heiman ML. GH-Releasing Peptide-2 Increases Fat Mass in Mice Lacking NPY: Indication for a Crucial Mediating Role of Hypothalamic Agouti-Related Protein. *Endocrinology*. 2002;143(2):558-568.

S34. Zou Y, Tang X, Yang S, et al. New insights into the function of the NLRP3 inflammasome in sarcopenia: mechanism and therapeutic strategies. *Metabolism*. 2024;158:155972.

S35. Huang HH, Wang TY, Yao SF, et al. Gastric Mobility and Gastrointestinal Hormones in Older Patients with Sarcopenia. *Nutrients*. 2022;14(9):1897.

S36. Wu Q, Chen J, Hua T, Cai J. Alpha-Melanocyte-Stimulating Hormone-Mediated Appetite Regulation in the Central Nervous System. *Neuroendocrinology*. 2023;113(9):885-904.

S37. Takahashi Y, Fujita H, Seino Y, et al. Gastric inhibitory polypeptide receptor antagonism suppresses intramuscular adipose tissue accumulation and ameliorates sarcopenia. *J Cachexia Sarcopenia Muscle*. 2023;14(6):2703-2718.

S38. Candemir B, İleri İ, Yalçın MM, et al. Relationship Between Appetite-Related Peptides and Frailty in Older Adults. *Endocr Res*. 2023;48(2-3):35-43.

S39. Sartori R, Milan G, Patron M, et al. Smad2 and 3 transcription factors control muscle mass in adulthood. *American Journal of Physiology-Cell Physiology*. 2009;296(6):C1248-C1257.

S40. Saitoh M, Takayama K, Hitachi K, et al. Discovery of a follistatin-derived myostatin inhibitory peptide. *Bioorg Med Chem Lett*. 2020;30(3):126892.

S41. Takayama K, Hitachi K, Okamoto H, et al. Development of Myostatin Inhibitory <scp>d</scp> -Peptides to Enhance the Potency, Increasing Skeletal Muscle Mass in Mice. *ACS Med Chem Lett*. 2022;13(3):492-498.

S42. Kim JH, Kim JH, Sutikno LA, et al. Identification of the minimum region of flatfish myostatin propeptide (Pep45-65) for myostatin inhibition and its potential to enhance muscle growth and performance in animals. *PLoS One*. 2019;14(4):e0215298.

S43. Ochi A, Abe T, Nakao R, et al. N-myristoylated ubiquitin ligase Cbl-b inhibitor prevents on glucocorticoid-induced atrophy in mouse skeletal muscle. *Arch Biochem Biophys*. 2015;570:23-31.

S44. Gao S, McMillan RP, Zhu Q, Lopaschuk GD, Hulver MW, Butler AA. Therapeutic effects of adropin on glucose tolerance and substrate utilization in diet-induced obese mice with insulin resistance. *Mol Metab*. 2015;4(4):310-324.

S45. Strickland I, Ghosh S. Use of cell permeable NBD peptides for suppression of inflammation. *Ann Rheum Dis*. 2006;65(suppl_3):iii75-iii82.

S46. Boso D, Maghin E, Carraro E, Giagante M, Pavan P, Piccoli M. Extracellular Matrix-Derived Hydrogels as Biomaterial for Different Skeletal Muscle Tissue Replacements. *Materials 2020, Vol 13, Page 2483*. 2020;13(11):2483.

S47. Kim CE, Shin SK, Bae HR, Su Lee J, Chul Shin Y, Kwon EY. The effect of granulated collagen tripeptide on sarcopenia in aged mice compared with collagen tripeptide. *J Funct Foods*. 2024;119:106318.

S48. Nedergaard A, Sun S, Karsdal MA, et al. Type VI collagen turnover‐related peptides—novel serological biomarkers of muscle mass and anabolic response to loading in young men. *J Cachexia Sarcopenia Muscle*. 2013;4(4):267-275.

S49. Kal S, Mahata S, Jati S, Mahata SK. Mitochondrial-derived peptides: Antidiabetic functions and evolutionary perspectives. *Peptides (NY)*. 2024;172.

S50. Lee C, Zeng J, Drew BG, et al. The Mitochondrial-Derived Peptide MOTS-c Promotes Metabolic Homeostasis and Reduces Obesity and Insulin Resistance. *Cell Metab*. 2015;21(3):443-454.

S51. Kumagai H, Coelho AR, Wan J, et al. MOTS-c reduces myostatin and muscle atrophy signaling. *American Journal of Physiology-Endocrinology and Metabolism*. 2021;320(4):E680-E690.

S52. Kim SJ, Guerrero N, Wassef G, et al. The mitochondrial-derived peptide humanin activates the ERK1/2, AKT, and STAT3 signaling pathways and has age-dependent signaling differences in the hippocampus. *Oncotarget*. 2016;7(30):46899-46912.

S53. Kim SJ, Devgan A, Miller B, et al. Humanin-induced autophagy plays important roles in skeletal muscle function and lifespan extension. *Biochimica et Biophysica Acta (BBA) - General Subjects*. 2022;1866(1):130017.

S54. Birk A V., Liu S, Soong Y, et al. The Mitochondrial-Targeted Compound SS-31 Re-Energizes Ischemic Mitochondria by Interacting with Cardiolipin. *Journal of the American Society of Nephrology*. 2013;24(8):1250-1261.

S55. Cai J, Xing L, Zhang W, Zhang J, Zhou L, Wang Z. Effect of Yeast-Derived Peptides on Skeletal Muscle Function and Exercise-Induced Fatigue in C2C12 Myotube Cells and ICR Mice. *J Agric Food Chem*. 2023;71(42):15522-15537.

S56. De Spiegeleer A, Wynendaele E, Descamps A, et al. The bacterial quorum sensing peptide iAM373 is a novel inducer of sarcopenia. *Clin Transl Med*. 2022;12(10).

S57. De Spiegeleer A, Descamps A, Wynendaele E, et al. Streptococcal quorum sensing peptide CSP-7 contributes to muscle inflammation and wasting. *Biochimica et Biophysica Acta (BBA) - Molecular Basis of Disease*. 2024;1870(4):167094.

S58. Masson SWC, Simpson RC, Cutler HB, et al. Genetic variance in the murine defensin locus modulates glucose homeostasis. Published online July 26, 2024.

S59. Palus S, Elkina Y, Braun T, et al. The erythropoietin‐derived peptide ARA 284 reduces tissue wasting and improves survival in a rat model of cancer cachexia. *J Cachexia Sarcopenia Muscle*. 2022;13(4):2202-2210.

S60. Mera P, Laue K, Wei J, Berger JM, Karsenty G. Osteocalcin is necessary and sufficient to maintain muscle mass in older mice. *Mol Metab*. 2016;5(10):1042-1047.

S61. Irintchev A, Wu MM, Lee HJ, et al. Glycomimetic Improves Recovery after Femoral Injury in a Non-Human Primate. *J Neurotrauma*. 2011;28(7):1295-1306.

S62. Brent MB, Thomsen JS, Brüel A. Short-term glucocorticoid excess blunts abaloparatide-induced increase in femoral bone mass and strength in mice. *Sci Rep*. 2021;11(1):12258.
